# Transient reprogramming of postnatal cardiomyocytes to a dedifferentiated state

**DOI:** 10.1371/journal.pone.0251054

**Published:** 2021-05-05

**Authors:** Thomas Kisby, Irene de Lázaro, Maria Stylianou, Giulio Cossu, Kostas Kostarelos

**Affiliations:** 1 Nanomedicine Lab, Faculty of Biology, Medicine and Health, The University of Manchester, Manchester, United Kingdom; 2 Division of Cell Matrix Biology and Regenerative Medicine, Faculty of Biology, Medicine and Health, The University of Manchester, Manchester, United Kingdom; 3 Catalan Institute of Nanoscience and Nanotechnology (ICN2), UAB Campus Bellaterra, Barcelona, Spain; University of Minnesota Medical School, UNITED STATES

## Abstract

In contrast to mammals, lower vertebrates are capable of extraordinary myocardial regeneration thanks to the ability of their cardiomyocytes to undergo transient dedifferentiation and proliferation. Somatic cells can be temporarily reprogrammed to a proliferative, dedifferentiated state through forced expression of *Oct3/4*, *Sox2*, *Klf4* and *c-Myc* (OSKM). Here, we aimed to induce transient reprogramming of mammalian cardiomyocytes *in vitro* utilising an OSKM-encoding non-integrating vector. Reprogramming factor expression in postnatal rat and mouse cardiomyocytes triggered rapid but limited cell dedifferentiation. Concomitantly, a significant increase in cell viability, cell cycle related gene expression and Ki67 positive cells was observed consistent with an enhanced cell cycle activation. The transient nature of this partial reprogramming was confirmed as cardiomyocyte-specific cell morphology, gene expression and contractile activity were spontaneously recovered by day 15 after viral transduction. This study provides the first evidence that adenoviral OSKM delivery can induce partial reprogramming of postnatal cardiomyocytes. Therefore, adenoviral mediated transient reprogramming could be a novel and feasible strategy to recapitulate the regenerative mechanisms of lower vertebrates.

## Introduction

The majority of cardiomyocytes in the mammalian heart exit the cell cycle soon after birth [1, 2]. Although evidence exists for the renewal of a small percentage of cardiomyocytes throughout the lifetime of mammals [3–5], such low rates cannot effectively support myocardial regeneration following an ischemic insult. In contrast, zebrafish have a much greater capacity for cardiomyocyte replenishment following various forms of cardiac injury [6–8]. Lineage tracing studies in these organisms have confirmed that pre-existing cardiomyocytes are the primary source of regenerated myocardium, indicating that extensive cardiomyocyte proliferation takes place after injury [9]. Additionally, it has been observed that cardiomyocyte cell cycle re-entry coincides with partial dedifferentiation to a progenitor-like state [10–14]. A similar albeit more subtle response is observed in embryonic and neonatal mammals however, this regenerative capacity is quickly lost within the first few days after birth [15–17]. Inducing limited dedifferentiation of postnatal mammalian cardiomyocytes could therefore be an attractive strategy to enhance the proliferative capacity of these cells.

A defined combination of transcription factors; *Oct3/4*, *Sox2*, *Klf4* and *cMyc* (OSKM), is thought to be universal in its ability to reset the epigenetic modulations that maintain cells in their differentiated state [18]. Indeed, many cell types including cardiomyocytes have been completely reprogrammed to an embryonic stem cell (ESC)-like state, known as induced pluripotent stem cells (iPSCs), through the forced expression of these factors [19–21]. To reach *bona fide* iPSCs, OSKM overexpression needs to be maintained for at least 8–12 days [22, 23]. If OSKM expression is withdrawn earlier, cells reach instead a state of partial or incomplete reprogramming that retain transcriptional and epigenetic marks from the cell type of origin [24–26]. The spontaneous re-differentiation of partially reprogrammed cells to their cell type of origin following OSKM withdrawal has been frequently reported [22, 23, 27, 28]. For such reasons, partially reprogrammed cells are increasingly recognised as potential novel sources of lineage restricted progenitor-like cells for cell therapy applications [24, 29].

While partial and transient OSKM mediated reprogramming of several cell types has been demonstrated, the response of cardiomyocytes to short term OSKM expression is yet to be explored. Furthermore, previous investigations have largely relied on transgenic cell inducible models limiting their *in vivo* translatability. Here, we aimed to understand whether cardiomyocytes could be partially reprogrammed *in vitro* using adenoviral mediated OSKM expression. We further investigated the proliferative capacity of reprogrammed cardiomyocytes and explored their propensity to return to their original phenotype in the absence of additional differentiation stimuli.

## Results

### Efficient expression of OSKM in cardiomyocytes using a polycistronic adenoviral vector

**Fig 1:**
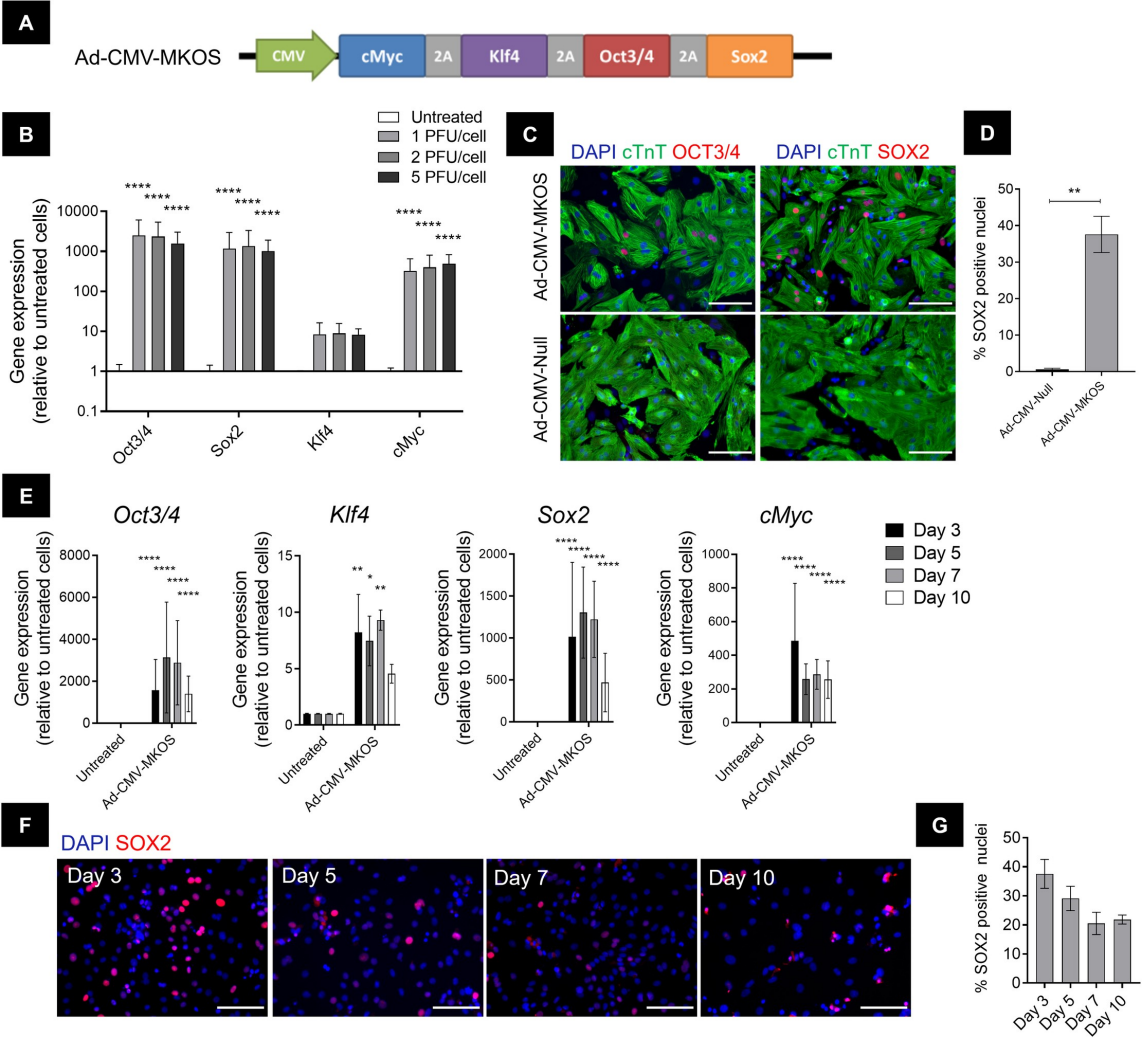
OSKM is efficiently delivered to postnatal cardiomyocytes using a polycistronic adenoviral vector. (**A**) Schematic of Ad-CMV-MKOS expression cassette. (**B**) Gene expression of total *OSKM* relative to untreated cells 3 days post transduction with Ad-CMV-MKOS at MOIs 1–5 PFU/cell (n = 3). (**C**) Representative images of OCT3/4 and SOX2 protein expression in cardiomyocytes stained with cTnT (scale bar = 100 μm). (**D**) Transduction efficiency expressed as percentage nuclei positive for SOX2 (n = 3 replicates/4-6 fields per replicate). (**E**) Timecourse of total *OSKM* gene expression relative to untreated cardiomyocytes (n = 3). (**F**) Representative images of Ad-CMV-MKOS transduced cells on days 3–10 post transduction (scale bars = 100 μm). (**G**) Quantification of the SOX2 positive nuclei in Ad-CMV-MKOS transduced cardiomyocytes (n = 3 replicates/4-6 fields per replicate). (**B**) and (**E**) one-way ANOVA with Tukey’s post hoc analysis, (**D**) Unpaired t-test, *, **, and **** denote p<0.05, p<0.01 and p<0.0001, respectively.

To investigate the response of cardiomyocytes to OSKM expression we utilized primary cardiomyocytes extracted from postnatal day 2 Sprague Dawley rats. The purity of these cultures at the time of transduction was >80% as determined by cardiac troponin-T (cTnT) and NKX2-5 expression (**S1A–S1C Fig**), which is comparable with other reports [30]. To force the expression of OSKM in these cells and interrogate partial reprogramming, we used an adenoviral vector (Ad-CMV-MKOS) containing all four reprogramming genes in a single polycistronic expression cassette (**Fig 1A**). We hypothesised that, based on their non-integrating nature, adenoviral vectors would provide temporary OSKM expression compatible with partial cell reprogramming, as opposed to the sustained expression achieved with integrating viral vectors. We first investigated relative mRNA expression of total *Oct3/4*, *Sox2*, *Klf4* and *cMyc* 3 days post transduction with Ad-CMV-MKOS (**Fig 1B**). All four genes were overexpressed in cardiomyocytes by relatively low multiplicities of infection (MOIs), consistent with reports of the high amenability of this cell type to adenoviral mediated gene delivery [30, 31]. Protein expression of OCT3/4 and SOX2 was also confirmed by immunocytochemistry at this same timepoint (**Fig 1C and 1D**). An empty adenoviral vector (Ad-CMV-Null) was used throughout our study to ensure that changes observed were due to OSKM expression and not due to viral infection alone. Indeed, we did not observe the presence of OCT3/4+ or SOX2+ cells in Ad-CMV-Null treated cultures (**Fig 1C and 1D**). We next followed the expression of reprogramming factors over time. Adenoviral mediated *OSKM* expression peaked 3–5 days post transduction however, this level of expression was not sustained (**Fig 1E**). Similar expression kinetics were observed at the protein level with the highest number of SOX2+ cells observed 3 days after transduction (**Fig 1F and 1G**). Therefore, we focused on these timepoints to understand the early responses of cardiomyocytes to OSKM overexpression.

### OSKM overexpression drives rapid dedifferentiation of postnatal cardiomyocytes

**Fig 2:**
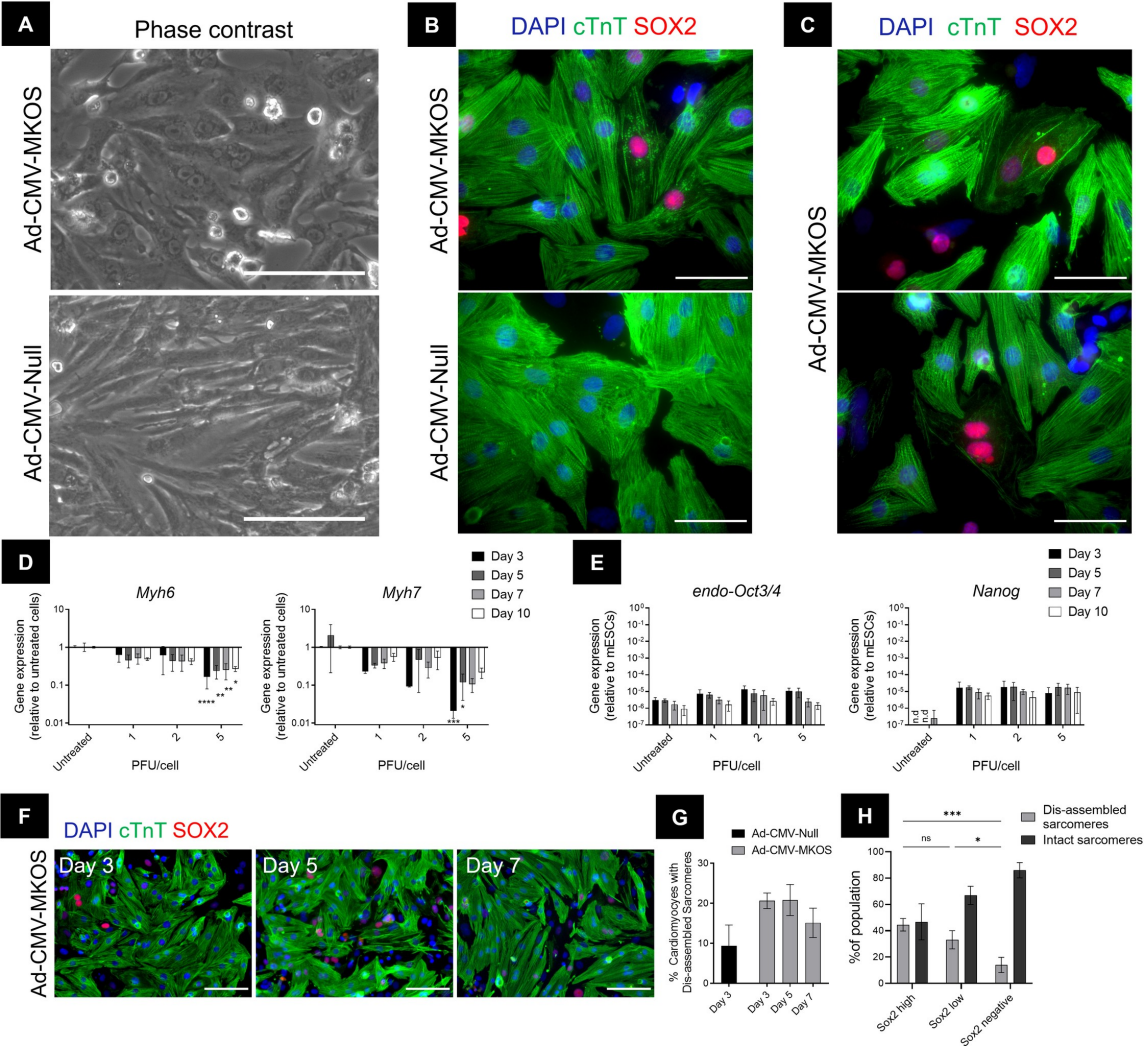
Overexpression of OSKM induces dedifferentiation of cardiomyocytes. (**A**) Representative phase contrast image of cardiomyocytes 3 days post transduction with Ad-CMV-MKOS or Ad-CMV-Null (scale bar = 100 μm) (n = 5 replicates/3 fields per replicate). (**B**) Immunofluorescence of OSKM induced sarcomere disassembly (scale bar = 50 μm). (**C**) Reduction in cTnT expression in SOX2 positive cardiomyocytes (scale bar = 50 μm). (**D**) Gene expression of *Myh6* and *Myh7* after exposure to 1–5 PFU/cell Ad-CMV-MKOS (n = 3). (**E**) Gene expression of *endo-Oct3/4* and *Nanog* relative to mouse ESCs (n.d denotes transcript not detected) (n = 3). (**F**) Immunofluorescence of cardiomyocyte morphology overtime following exposure to Ad-CMV-MKOS (scale bar = 100 μm). (**G**) Quantification of the percentage of cTnT positive cells showing disorganized sarcomere structure (n = 3 replicates/4-6 fields per replicate). (**H**) Quantification of the percentage of dedifferentiated cardiomyocytes in the SOX2 high, SOX2 low and SOX2 negative populations 3 days after transduction with Ad-CMV-MKOS (n = 4 replicates, 4–6 fields per replicate). (**D**-**E**) one-way ANOVA with Tukey’s post hoc, (**H**) two-way ANOVA with Tukey’s post hoc *, **, ***, and **** denote p<0.05, p<0.01, p<0.001, and p<0.0001, respectively.

Three days post transduction we observed substantial morphological and functional changes in Ad-CMV-MKOS transduced cardiomyocytes consistent with dedifferentiation [14]. OSKM transduced cardiomyocytes showed a flattened, less striated appearance with more prominent nuclei and nucleoli (**Fig 2A**). We also observed the cessation of autorhythmic contractile activity in the cells which possessed this altered morphology (**S1** and **S2 Videos**). Such morphological changes were further investigated by immunocytochemistry. Disassembly of sarcomeres within SOX2+ cardiomyocytes was clearly observable however, this was largely absent from Ad-CMV-Null treated cultures (**Fig 2B**). In addition, a visible reduction in cTnT expression was also apparent in a small number of OSKM transduced cardiomyocytes, indicating not only disassembly but also a reduction in the expression of sarcomere proteins (**Fig 2C**). Both phenomena recapitulate events characteristic of cardiomyocyte dedifferentiation in the zebrafish and newt regenerating myocardium [9, 10, 32]. *Myh6* and *Myh7* are genes which encode α- and β- myosin heavy chain (MHC) respectively which are essential proteins involved in the contractile functions of adult and neonatal ventricular myocytes [33]. The expression of such gene transcripts was downregulated in OSKM transduced cultures (**Fig 2D**), but remained unaltered in cardiomyocytes treated with the control vector confirming this response to be dependent on OSKM expression (**S2A Fig**). The downregulation of *Myh6* and *Myh7* occurred rapidly by day 3 post transduction and did not further decrease after this timepoint (**Fig 2D**). Additionally, expression of *endogenous-Oct3/4* and *Nanog* was not significantly different relative to control and their expression levels remained significantly lower than that observed in a mouse ESC line throughout this investigation (**Fig 2E**). In agreement with these observations, the de-differentiation of cardiomyocytes, which we characterised by the presence of a disassembled or absent sarcomere structure [14, 34] (**Fig 2B and 2C**) and loss of cTnT expression, appeared most prominent on days 3–5 post transduction coinciding with the peak in exogenous OSKM expression (**Fig 2F and 2G**). We also investigated whether the dedifferentiation of cardiomyocytes could be correlated with the level of SOX2 protein expression. We focussed on Ad-CMV-MKOS transduced cardiomyocytes and quantified the percentage of cardiomyocytes with dis-assembled sarcomeres in the SOX2 high, SOX2 low and SOX2 negative population on day 3 after transduction (**S3 Fig** and **Fig 2H**). While the percentage of dedifferentiated cardiomyocytes did not differ significantly in the SOX2 high (44.6 ± 4.8%) compared to the SOX2 low population (33.1 ± 6.9%), both showed significantly more dedifferentiated cells than the SOX2 negative cardiomyocytes further confirming this response to be OSKM dependent. Notably on day 10, the striated cardiomyocyte-like morphology was discernible and autorhythmic contractile activity was partially restored in these cells (**S3** and **S4 Videos**). Overall, these data suggest that postnatal cardiomyocytes can undergo OSKM-mediated dedifferentiation in line with the early stages of cell reprogramming.

To confirm the cardiomyocyte origin of OSKM transduced cells with reduced or absent cTnT expression, we utilized postnatal mouse cardiomyocytes from an αMHC-Cre-tdTomato lineage tracing model [35]. Following the same transduction protocol utilized previously, we observed sarcomere disassembly and reduced cTnT expression in tdTomato positive cells 3 days after transduction with Ad-CMV-MKOS (**S4A Fig**). From this we also quantified the number of SOX2+tdTomato+ cells that did not express any discernible cTnT (**S4B Fig**). The presence of SOX2+tdTomato+ cells with reduced cTnT expression confirm our previous observations that adenoviral mediated OSKM expression enables postnatal cardiomyocyte dedifferentiation.

### Forced dedifferentiation of cardiomyocytes with OSKM increases cell cycle activity

**Fig 3:**
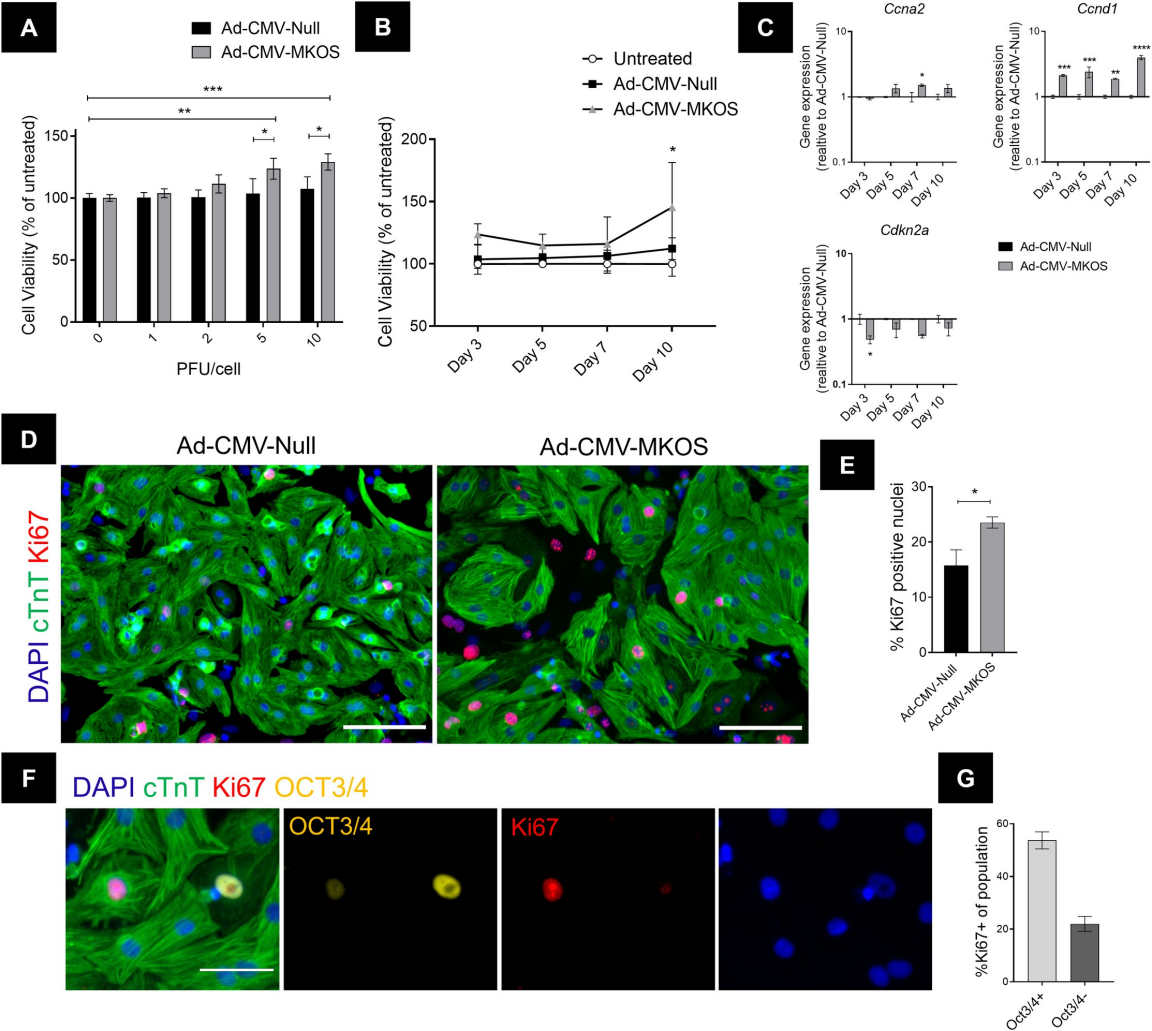
Forced dedifferentiation of cardiomyocytes by reprogramming enhances viability and cell cycle activity. (**A**) Relative cell viability 3 days post transduction with Ad-CMV-Null or Ad-CMV-MKOS at 1–10 PFU/cell (n = 3). (**B**) Timecourse of viability measurements at 5 PFU/cell (n = 3). (**C**) Expression of cell cycle related genes (n = 2). (**D**) Ki67 expression in cardiomyocytes 3 days post transduction (Scale bar = 100 μm). (**E**) Quantification of Ki67+ nuclei (n = 3 replicates/4-6 fields per replicate). (**F**) Colocalisation of Ki67 within dedifferentiating OCT3/4+ cardiomyocytes (Scale bar = 50 um). (**G**) Quantification of the percentage of Ki67+ cells in the OCT3/4+ and OCT3/4- population 3 days after transduction with Ad-CMV-MKOS (n = 3 replications, 4–6 fields per replicate). (**A**-**C**) two-way ANOVA with Tukey’s post hoc analysis, (**E**) Unpaired t-test. *, **, ***, and **** denote p<0.05, p<0.01, p<0.001, and p<0.0001, respectively.

It was next assessed whether OSKM induced dedifferentiation would enhance the capacity of postnatal cardiomyocytes to undergo proliferation. We first assessed this using an alamarBlue cell viability assay [36]. OSKM expression increased the relative viability of cardiomyocytes 3 days post transduction compared to cells treated with the control vector (**Fig 3A**) and this was maintained over time (**Fig 3B**). Next, we investigated the expression of cell cycle related genes under the assumption that this would be altered if partially reprogrammed cardiomyocytes re-entered the cell cycle. Following OSKM overexpression, we observed an increase in *CyclinA2* (*Ccna2*), *CyclinD1* (*Ccnd1*) expression concomitant with a downregulation of the negative cell cycle regulator *Cdkn2a* (**Fig 3C**). We next used Ki67 staining to assess the number of cells actively in the cell cycle 3 days post transduction. We observed a significant increase in the total percentage of Ki67+ nuclei in cardiomyocytes treated with Ad-CMV-MKOS compared to those treated with the control vector (**Fig 3D and 3E**). Given our previous observation that some cardiomyocytes lose cTnT expression following OSKM transduction, we concluded that this (cTnT expression) would not be a reliable indicator of the number of dedifferentiated cardiomyocytes undergoing proliferation. Instead, we utilized αMHC-Cre-tdTomato cardiomyocytes to confirm the source of proliferating cells and identified an increase in the percentage of Ki67+tdTomato+ cells that was consistent with that observed for total Ki67+ nuclei (**S5A and S5B Fig**). As expected, many Ki67+tdTomato+ cells showed reduced or absent cTnT expression (**S5C Fig**). Furthermore, in agreement with the hypothesis that OSKM mediated dedifferentiation drives cell cycle activation, co-localisation of Ki67 and OCT3/4 were observed within cardiomyocytes that showed this dedifferentiated phenotype (**Fig 3F**). In the Ad-CMV-MKOS treated cells a higher proportion of Ki67+ cells were identified in the OCT3/4+ population (53.7 ± 3.2%) than the OCT3/4- population (22.0 ± 2.8%) (**Fig 3G**). As sarcomere disassembly occurs during progression through the cell cycle and mitosis [37] it is likely that this effect on cardiomyocyte sarcomere appearance is also influenced by the enhanced cell cycle activity induced by OSKM. Overall, these data suggest that forced OSKM expression in postnatal cardiomyocytes enhances their cell cycle activity by inducing cellular dedifferentiation.

### Cardiomyocytes are partially but not completely reprogrammed by adenoviral mediated OSKM expression

**Fig 4:**
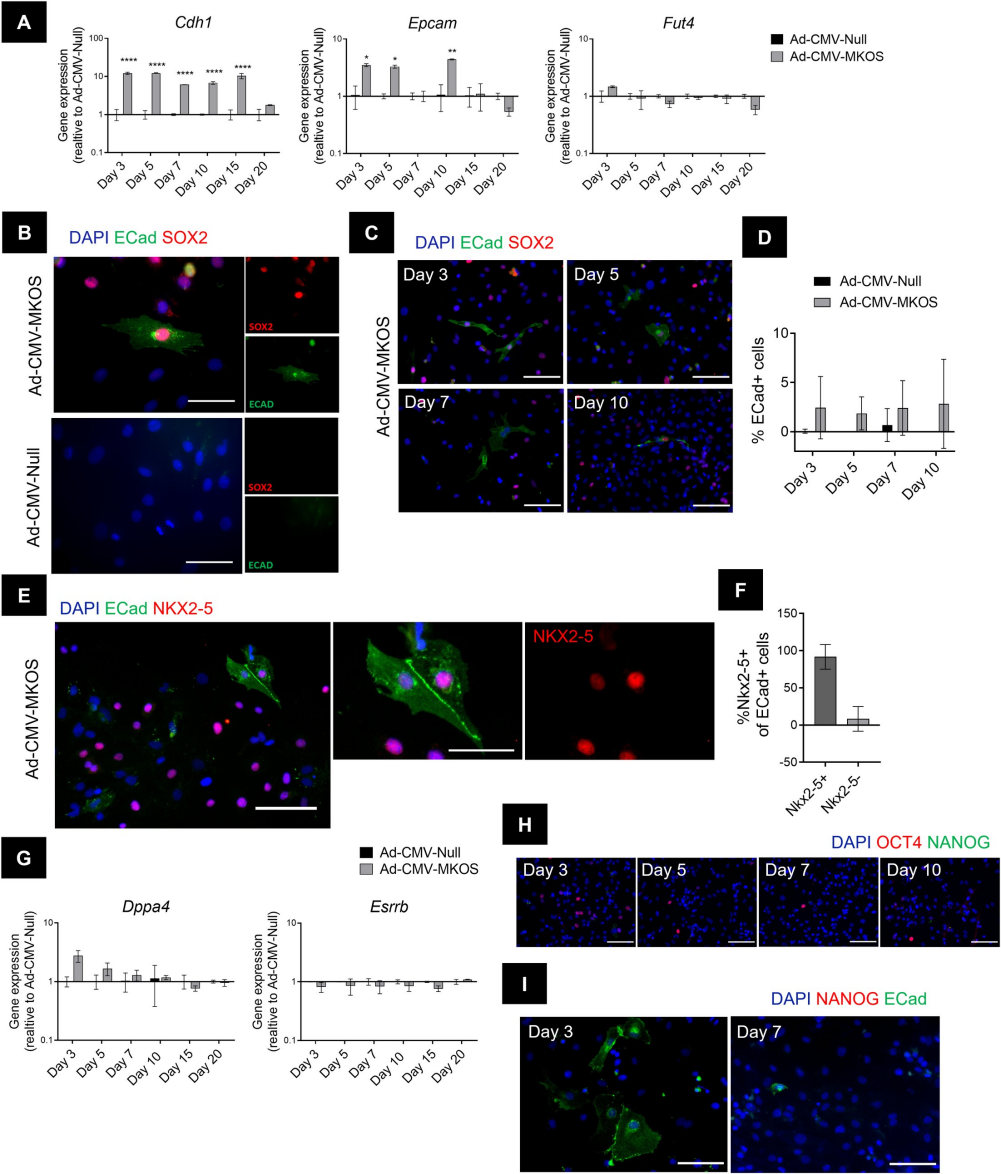
Cardiomyocytes are partially but not completely reprogrammed by adenoviral mediated OSKM expression. (**A**) Gene expression of genes associated with early stages of cell reprogramming to pluripotency (n = 2) (**B**) Presence of ECad+ cells in Ad-CMV-MKOS treated cultures day 3 post transduction (Scale bar = 50 μm). (**C**) Ecad+ cells present at different timepoints in Ad-CMV-MKOS transduced cardiomyocytes (Scale bar = 100 μm). (**D**) Quantification of percentage of ECad positive cells at each timepoint (n = 3 replicates/3-4 fields per replicate). (**E**) Coexpression of NKX2-5 in ECad positive cells (Scale bars = 100 μm and 50 μm). (**F**) Quantification of percentage of ECad+ cells co-expressing NKX2-5 (n = 2 replicates, 4 fields per replicate). (**G**) Expression of pluripotency genes following Ad-CMV-MKOS transduction (n = 2). (**H**) Absence of NANOG expression in Ad-CMV-MKOS treated cardiomyocytes over 10 days (scale bar = 100 μm) (n = 3 replicates/4-6 fields per replicate). (**I**) Absence of NANOG expression in ECad positive cells derived from cardiomyocytes (scale bar = 100 μm) (n = 2 replicates per 4–6 fields per replicate). (**A**), (**D**) and (**G**) two-way ANOVA with Tukey’s post-hoc analysis, *, **, and **** denotes p<0.05, p<0.01 and p<0.0001 respectively.

We next investigated the extent of cardiomyocyte reprogramming achieved by a single administration of the OSKM expressing adenoviral vector. In these experiments, we extended the timepoint of investigation up to 20 days post transduction, which is comparable to the length of conventional iPSC generation protocols [18, 38]. Mesenchymal to epithelial transition (MET) is a key event in the early stages of reprogramming [23], during which stemness-associated makers including SSEA1 (a carbohydrate ESC marker that is a product of *Fut4*) are induced [39]. As early as 3 days after OSKM transduction, we observed an upregulation in epithelial associated genes *Cdh1* and *Epcam* in cardiomyocytes treated with OSKM (**Fig 4A**). In contrast, no significant increases in the stem cell marker *Fut4* was detected at any of the timepoints investigated (**Fig 4A**). To confirm the increase in epithelial markers we stained cells for E-Cadherin (ECad) and identified a small number of ECad+ cells specifically in the OSKM treated cultures (**Fig 4B**). Consistent with *Cdh1* gene expression, the number of ECad+ cells did not increase over the timepoints investigated (**Fig 4C and 4D**). Notably the percentage of ECad+ cells was substantially lower than the number of cells observed to be undergoing dedifferentiation (**Fig 4D**). Additionally, these cells did not appear to form cobblestone-like epithelial cell colonies suggesting a full transition to an epithelial phenotype had not taken place (**Fig 4C**). To determine the source of ECad+ cells, they were stained cells for the cardiac transcription factor NKX2-5 which is one of the earliest markers of the cardiac lineage [40]. On day 3 post transduction, the majority of ECad+ cells co-expressed NKX2-5 suggesting cardiomyocytes were the primary source of these cells (**Fig 4E and 4F, S6 Fig**). Furthermore, the maintenance of NKX2-5 expression in these cells further indicates that transduced cardiomyocytes do not pass completely through MET and still retain markers of their cardiomyocyte origin. To further confirm that Ad-CMV-MKOS transduction was able to induce partial MET in cardiomyocytes we used αMHC-Cre-tdTomato mouse cells. Consistent with the results for rat cardiomyocytes, ECad+ cells were identified specifically in Ad-CMV-MKOS transduced cultures (**S7A Fig**). These ECad+ cells were also tdTomato+ confirming that cardiomyocytes contribute to this population of partially reprogrammed cells (**S7B Fig**). Overall, these data suggest that a small number of cardiomyocytes initiate MET in line with the earliest stages of somatic cell reprogramming [23].

We also investigated the expression of genes associated with later stages of reprogramming to pluripotency including *Esrrb* and *Dppa4* [23, 25]. Both these genes are members of the core pluripotency gene regulatory network and are upregulated during maturation and stabilisation respectively [41, 42]. Following OSKM transduction, we observed a transient increase in *Dppa4* gene expression (**Fig 4G**). However, the upregulation of this transcript was not sustained over time and we did not observe significant increases in the mRNA levels of *Esrrb* (**Fig 4G**). These results suggest that a complete pluripotency transcriptional program was not established. In addition, we did not detect NANOG protein expression at any of the time points investigated (**Fig 4H**) even when we specifically interrogated NANOG expression in cells that had initiated the MET process (**Fig 4I**). Overall, these data confirm that reprogramming of cardiomyocytes transduced with Ad-CMV-MKOS is incomplete at least up to day 20 post transduction.

### Adenoviral mediated reprogramming of postnatal cardiomyocytes is transient

**Fig 5:**
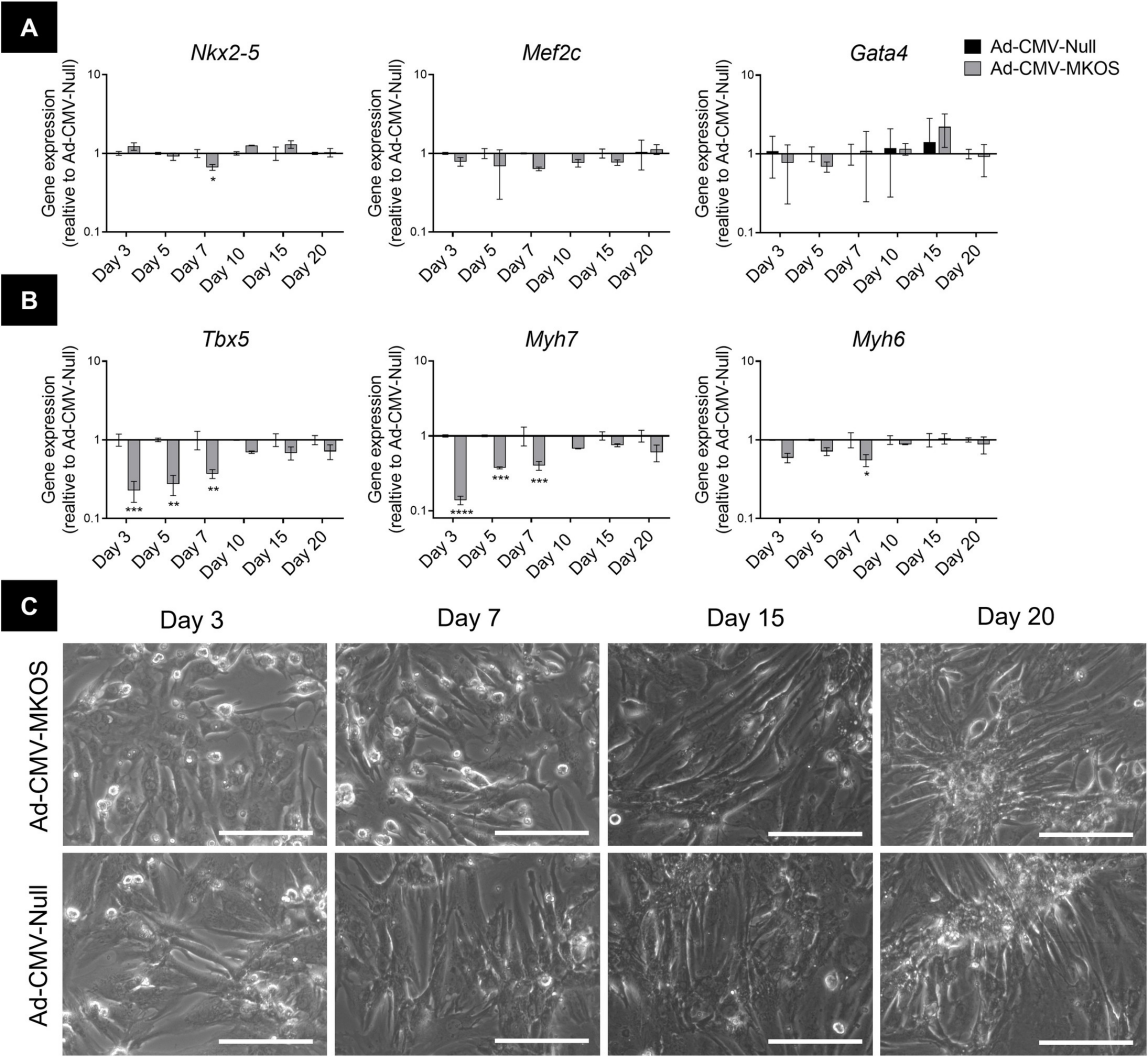
Adenoviral mediated reprogramming of postnatal cardiomyocytes is transient. (**A**) Expression of cardiogenic transcription factors after transduction with Ad-CMV-MKOS or Ad-CMV-Null (n = 2). (**B**) Transient downregulation of cardiomyocyte related genes (n = 2). (**C**) Phase contrast microscopy of cardiomyocytes following transduction with Ad-CMV-MKOS or Ad-CMV-Null (Scale bar = 100 μm). Representative images from n = 3 replicates/3 fields per replicate. (**A**) and (**B**) two-way ANOVA with Tukey’s post-hoc analysis, *, **, *** and **** denotes p<0.05, p<0.01, p<0.001 and p<0.0001 respectively.

Consistent with the hypothesis that cardiomyocytes are only partially reprogrammed by transient OSKM expression, we did not observe substantial changes in the expression of cardiogenic transcription factors *Nkx2-5*, *Mef2c* or *Gata4* indicating they are conserved in OSKM transduced cardiomyocytes (**Fig 5A**). However, we did observe transient decreases in the expression of *Tbx5* and cardiomyocyte contractile genes *Myh7* and *Myh6* (**Fig 5B**). The expression of these markers was at its lowest on day 3 post transduction before gradually returning toward baseline levels thereafter. In agreement, the morphological differences (i.e. dedifferentiated phenotype) observed day 3 post transduction were no longer present at the later timepoints investigated (day 15–20) when cells were indistinguishable from control vector treated cardiomyocytes (**Fig 5C**). Furthermore, the beating capacity of the cardiomyocytes cultures was restored by day 10 post transduction and was maintained through to day 20, suggesting that partially reprogrammed cardiomyocytes regain their functionality and contractile properties (**S5** and **S6 Videos**). Importantly, we did not detect the generation of pluripotent stem cell-like colonies at any timepoint during these investigations (**Fig 5C**). This data suggests that, in the absence of additional stimuli, partially reprogrammed cardiomyocytes have the capacity to spontaneously regain a phenotype close to their original identity.

### Partial reprogramming of cardiomyocytes is enhanced in ESC culture media conditions

**Fig 6:**
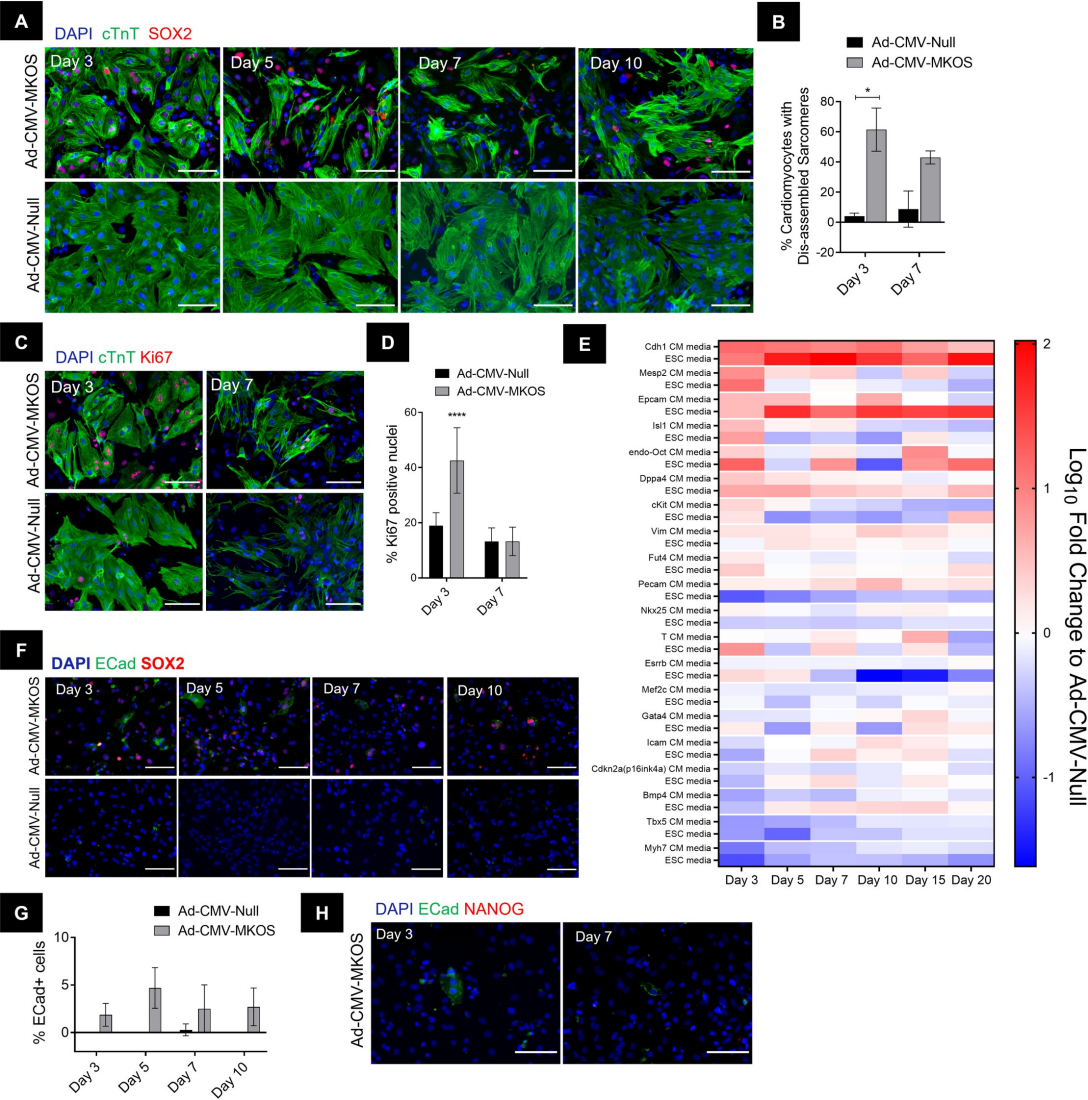
Partial reprogramming of cardiomyocytes is enhanced by additional media components. (**A**) Immunofluorescence staining of cardiomyocytes cultured in ESC media (Scale bar = 100 μm). (**B**) Quantification of cTnT positive cells showing sarcomere dis-assembly (n = 2 replicates/4-6 fields per replicate). (**C**) Immunostaining of Ki67 in transduced cardiomyocytes in ESC media (scale bar = 100 μm). (**D**) Quantification of percentage of Ki67+ cells (n = 2 replicates/4-6 fields per replicate). (**E**) RT-qPCR analysis of changes in gene expression relative to Ad-CMV-Null transduced cells in standard cardiomyocyte (CM) or ESC specified media (n = 2). (**F**) Presence of ECad+ cells generated from cardiomyocytes transduced with Ad-CMV-MKOS (Scale bar = 100 μm). (**G**) Quantification of ECad positive cells (n = 2 replicates, 4–6 fields per replicate). (**H**) Absence of NANOG expression in ECad positive cells derived from cardiomyocytes (scale bar = 100 μm). (n = 2 replicates/4-6 fields per replicate). (**B**), (**D**) and (**G**) two-way ANOVA with Tukey’s post-hoc analysis. * and **** denote p<0.05 and p<0.0001 respectively.

Supplementation of cell culture medium with cytokines such as leukaemia inhibitory factor (LIF) is required for the generation and maintenance of rodent pluripotent cells [43]. We next investigated if culturing Ad-CMV-MKOS transduced cardiomyocytes in ESC culture medium containing LIF would enable the further progression of reprogramming toward pluripotency. Cardiomyocytes cultures were switched to these culture conditions 2 days after viral transduction and maintained with daily media changes thereafter. As in our previous observations in cardiomyocyte (CM) media, we observed indications of dedifferentiation such as sarcomere disassembly, reduced cTnT expression, and an increase in the cTnT negative population of cardiomyocytes (**Fig 6A**). This is further evidenced by a more heterogeneous and high intensity fluorescence signal corresponding to the cTnT staining indicating that this protein is no longer evenly distributed in organised sarcomeres as it is in the control treated cells [34]. Notably, the percentage of cardiomyocytes showing sarcomere dis-assembly was substantially greater than that previously observed in basal media indicating more efficient induction of partial dedifferentiation (**Fig 6B**). In agreement with this we also observed a more robust increase in the percentage of Ki67+ cells 3 days following OSKM transduction (**Fig 6C and 6D**). However, this difference was no longer present by day 7, suggesting that the increase in cell cycle activity was transient (**Fig 6D**). We next compared the gene expression profiles of Ad-CMV-MKOS transduced cardiomyocytes maintained in CM or ESC culture medium. ESC culture conditions both enhanced and prolonged the reprogramming effects triggered by OSKM as indicated by the expression of epithelial (*Cdh1*, *Epcam*) and cardiomyocyte (*Myh7*, *Tbx5*, *Nkx2-5*) related genes (**Fig 6E**). However, the expression of late stage reprogramming genes *Dppa4* and *Esrrb* were not increased further by this specialised media, suggesting cells were still unable to reach the later stages of reprogramming to pluripotency (**Fig 6E**). We again observed the presence of ECad+ cells however, as observed for cardiomyocyte media, the number of these cells did not appear to increase substantially over the timepoints investigated (**Fig 6F and 6G**). Furthermore, we were unable to detect NANOG protein expression, including in the cells which had undergone MET (**Fig 6H**). Finally, no pluripotent stem cell-like colonies were identified for up to 20 days after Ad-CMV-MKOS transduction (**S8A and S8B Fig**), confirming that cell reprogramming mediated by a single transduction of a non-integrating adenoviral vector is incomplete. Together, these results indicate that media conditions are not the limiting factor on the level of reprogramming reached by cardiomyocytes which is instead limited by the transient nature of adenoviral mediated OSKM expression. However, media conditions do appear to play a role in the efficiency and the re-differentiation of partially reprogrammed cardiomyocytes.

## Discussion

Here we demonstrate that postnatal mammalian cardiomyocytes can be partially and transiently reprogrammed to a dedifferentiated state through adenoviral mediated OSKM expression. We chose an adenoviral vector to investigate partial cardiomyocyte reprogramming based on their established ability to efficiently transduce cardiomyocytes at low MOIs without transgene integration [31]. Since proliferation is one of the early steps during cell reprogramming, we hypothesised that the OSKM transgene would be rapidly diluted in cells undergoing this process and fall below the levels required to establish and sustain pluripotency [22, 44]. Indeed, consistent with other investigations using adenoviral vectors to deliver OSKM, we did not observe the generation of pluripotent stem cells following a single adenoviral transduction [45–47]. However, the level and extent of reprogramming achieved in cells following a single adenoviral mediated delivery of an OSKM transgene had not been previously interrogated.

We confirmed the initiation of reprogramming as evidenced by cellular dedifferentiation and the induction of MET which is consistent with the responses observed following OSKM induction in other cell types [23, 28, 38, 44]. However, we did not observe NANOG+ cells arising from Ad-CMV-MKOS transduced cultures, nor the generation of pluripotent stem cell-like colonies confirming this reprogramming response to be incomplete. This is in agreement with other studies that have demonstrated the maturation phase, characterised by the establishment of pluripotency gene networks, acts as the major limiting step to the progression of reprogramming [28]. We also observed the maintenance of NKX2-5 expression in cardiomyocytes that had initiated MET. As Nkx2-5 is one of the earliest markers of cardiogenic differentiation its persistence in this case could indicate cells are still committed to a cardiac lineage [40, 48]. Indeed, the expression of this gene remains relatively unchanged in the regenerating zebrafish heart where transient downregulation of other cardiac genes is observed [7, 11]. Partial reprogramming of mammalian cardiomyocytes may therefore offer a method to recapitulate these limited dedifferentiation responses that contribute to the enhanced regenerative capacity of model organisms.

A single study has investigated the early stages of cardiomyocyte reprogramming to pluripotency utilising a doxycycline inducible OSKM expression system [21]. In this study the authors identified that early in the reprogramming process, prior to MET and complete loss of somatic cell identity, that genes associated with cell cycle progression are activated. Our results agree with this as both cell cycle genes were upregulated and an increase in the Ki67 positive proportion of cells was observed as early as day 3 post transduction. Furthermore, while a proportion of cells did appear to initiate MET, this was incomplete and less frequent than the number of cells undergoing dedifferentiation and proliferation. Interestingly, in the present study, adenoviral mediated reprogramming of cardiomyocytes was not only partial but also transient in nature. These results are in agreement with observations in other cell types that withdrawal of exogenous OSKM expression prior to the establishment of transgene independent pluripotency enables the reversion of cells to a phenotype close to that of the starting cell population [22–24, 27, 28]. The presence of epigenetic memory has been identified in functionally pluripotent iPSCs that can greatly influence their differentiation potential [19, 20, 49–52]. Therefore, it is unsurprising that in cases of partial reprogramming where the epigenome is even more conserved that cells would be further restricted toward their original lineage. However, future work is necessary to understand how the level of OSKM expression and stoichiometry of these factors correlates with the reprogramming response both in terms of level of dedifferentiation, induction and progression of the cell cycle and the fate of these cells at the single cell level.

Given the similarities between OSKM induced transient reprogramming presented here and the endogenous regenerative mechanisms observed in model organisms [6, 10, 11], transient OSKM expression could be a novel strategy to stimulate proliferation of cells in the injured myocardium *in vivo*. Some of us have previously demonstrated that somatic cells can be transiently reprogrammed *in vivo* through non-integrating vector mediated expression of OSKM without leading to teratoma or tumorigenesis [53–57]. Ourselves and others have also demonstrated that transient *in vivo* reprogramming can be used to enhance the regeneration of injured tissue [56, 58, 59] and rejuvenate aged tissues [60] without leading to teratoma generation. The present study demonstrates that transient OSKM expression in cardiomyocytes *in vitro* enables a temporary passage through a proliferative de-differentiated state which is encouraging for the potential applications of this approach in the heart. Furthermore, in contrast to investigations relying on inducible transgenic models [58, 60], the use of an adenoviral vector to induce transient reprogramming offers a more readily translatable approach for *in vivo* applications.

## Conclusion

This study offers previously unreported demonstration of adenoviral-mediated OSKM expression to enable transient dedifferentiation of primary cardiomyocytes. This, in many ways, recapitulates what is seen during lower vertebrate and neonatal mammalian regeneration. We therefore propose that it could be utilised further as an investigative tool to inform cardiac regenerative strategies that aim to enhance the proliferative capacity of cardiomyocytes *in situ*.

## Materials and methods

### Animals

All animal experiments were performed with prior approval from the UK Home Office under the project license (P089E2E0A) and in compliance with the UK Animals (Scientific Procedures) Act, 1986. Time mated Sprague-Dawley rats were obtained from Charles River (UK) and allowed at least 1 week to acclimatise before litters were born. αMHC-Cre-tdTomato (C57BL6/129/FVB) mice were derived from crossing αMHC-Cre (B6.FVB-Tg(Myh6-cre)2182Mds/J, JAX stock 011038) [35] mice, kindly provided by Dr Elizabeth J. Cartwright (University of Manchester, UK) with B6.Cg-Gt(ROSA)^26Sortm14(CAG-tdTomato)Hze^/J tdTomato reporter mice (JAX stock 007914) [61]. The resultant mouse strain αMHC-Cre-tdTomato (C57BL6/129/FVB) was maintained as heterozygous for the αMHC-Cre transgene and homozygous for ROSA26tdTomato.

### Viral vectors

The viral vectors used in this study were purchased from Vector Biolabs (USA). Ad-CMV-MKOS is a Human Adenovirus type 5 (dE1/E3) containing the polycistronic expression cassette encoding mouse c-Myc-F2A-Klf4-T2A-Oct4-E2A-Sox2 under the control of a CMV promoter. A serotype matched adenovirus containing an empty CMV promoter expression cassette was used as a control. Vectors were provided in phosphate buffered saline (PBS) containing 5% glycerol and were diluted in culture media immediately prior to transduction.

### Primary postnatal rat cardiomyocyte extraction

Cardiomyocytes were extracted from 2 day old Sprague-Dawley (SD) rats (Charles River, UK) as described previously with some modifications [62]. Animals were culled by cervical dislocation followed by decapitation, hearts were excised and washed twice in ADS buffer (116 mM NaCl, 20 mM HEPES, 1 mM NaH_2_PO_4_, 5.5 mM glucose, 5.5 mM KCl and 1 mM MgSO_4_, pH 7.4). Ventricles were dissected from the atria and cut laterally. Next, they were digested in 7 ml ADS buffer containing 0.6 mg/ml collagenase A (Roche, Germany) and 0.6 mg/ml pancreatin (Sigma-Aldrich, UK) at 37 ^o^C for 5 minutes, under moderate shaking. Digested tissue was then passed several times through a 25 ml glass pipette with the resulting supernatant collected and passed through a 70 μm cell strainer (Corning, UK). Enzymatic digestion was inactivated with 3 ml fetal bovine serum (FBS) (ThermoFisher, UK). This process was repeated 9 times or until ventricular tissue had been digested completely. The resulting cell solution was centrifuged at 1200 rpm, re-suspended in pre-plating medium (68% Dulbecco’s Modified Eagle Medium (DMEM, Sigma-Aldrich, UK), 17% Medium-199 (M199, Sigma-Aldrich, UK) supplemented with 10% horse serum (ThermoFisher, UK), 5% FBS and 2.5 μg ml^-1^ amphotericin B (Sigma-Aldrich, UK)) and plated on 90 mm tissue culture dishes (Corning, UK). Non-myocytes were left to adhere for 1 hour and the cardiomyocyte enriched supernatant was then collected. The resulting cardiomyocytes were counted and plated at 0.2x10^6^ cells/well in Corning Primaria 24 well plates, 8x10^4^ cells/well in poly-l-lysine (Sigma-Aldrich, UK)/Laminin (Sigma-Aldrich, UK) coated MilliCell EZ 8 well chambers slides (Merck, UK) and 3x10^4^ cells/well in Corning Primaria 96 well plates. Cardiomyocytes were initially maintained in pre-plating medium with the addition of 100 μM BrdU (Sigma-Aldrich, UK) at 37 ^o^C for 24 hours to limit proliferation of non-myocytes [63]. Cells were then washed twice in warm PBS containing Ca^2+^ and Mg^2+^ (Gibco, UK) and medium replaced with cardiomyocyte maintenance medium (80% DMEM and 20% Medium 199 supplemented with 1% FBS, 2.5 μg ml^-1^ amphotericin B and 100 μM BrdU) in which cells were maintained until viral transduction.

### Adenoviral transduction and culturing

Cardiomyocytes were transduced with Ad-CMV-MKOS (Vector Biolabs) or Ad-CMV-Null (Vector Biolabs) at MOIs of 1–10 PFU/cell pre-diluted in cardiomyocyte maintenance media containing 2% FBS without BrdU. Unless otherwise noted in the figure or legend, cardiomyocytes were transduced with 5 PFU/cell. Cells were then maintained at 37°C for 24 hours before virus containing media was replaced with fresh cardiomyocyte maintenance media (without BrdU). Media was exchanged every other day thereafter. In a subset of experiments, as indicated in the text, media was replaced with ESC medium (Knockout DMEM/F12 (ThermoFisher, UK) supplemented with 15% Knockout Serum Replacement (ThermoFisher, UK), 1% StemPro non-essential amino acids (ThermoFisher, UK), 50 μM β-mercaptoethanol (Sigma-Aldrich, UK), 10 ng/ml of mouse leukaemia inhibitory factor (eBioscience, UK)) and was changed daily thereafter.

### Primary postnatal αMHC-Cre-tdTomato mouse cardiomyocytes

Transgenic cardiomyocytes were extracted from postnatal day 2 pups using a modified version of the protocol for rats. In brief, prior to tissue digestion and cell extraction, hearts were screened with an EVOS FL fluorescence microscope to select hearts that were positive for tdTomato fluorescence confirming a positive αMHC-Cre genotype. Following extraction, cardiomyocytes were counted and seeded at 8x10^4^ cells/well in poly-l-lysine/laminin coated MilliCell EZ 8 well chambers slides. Transduction and maintenance of mouse cardiomyocytes was performed as described above for rat cardiomyocytes.

### Imaging/Videos

Still phase contrast images were taken on an EVOS FL microscope using the 10x and 20x objective. Videos were captured on an Olympus IX83 inverted microscope using a 20x objective and captured using an Orca ER camera (Hamamatsu, UK) through MMI Cell tools software (MMI, Switzerland).

### alamarBlue assay

Cell viability was determined using an alamarBlue assay as previously described [36]. Resazurin sodium salt (Sigma-Aldrich, UK) was dissolved in PBS containing Ca^2+^ and Mg^2+^ which was further diluted in cell culture medium to a final concentration of 20 μg ml^-1^, immediately before adding to the cells in 96 well plates. Cells were then incubated for 2 hours at 37 ^o^C in the absence of light. Fluorescence was read directly from plates using a FLUOstar Omega (BMG Labtech, UK) plate reader with 544 nm excitation and 590 nm emission wavelengths. The percentage cell viability relative to untreated cells was calculated using the mean fluorescence intensity measurement from 6 replicates per condition.

### Reverse-transcription real-time quantitative polymerase chain reaction (RT-qPCR)

Cultured cells were lysed directly in tissue culture plates in lysis buffer (ThermoFisher, UK) containing 1% β-mercaptoethanol. RNA was then extracted from the cells or tissues using the PureLink RNA mini kit (ThermoFisher, UK) following the manufacturer’s guidelines. Eluted RNA was subjected to an additional DNase treatment using the RapidOut DNA Removal kit (ThermoFisher, UK) to remove any potentially contaminating viral DNA. Purified RNA was quantified using a biophotometer and 0.8–1 μg of RNA was used to produce cDNA using the High Capacity Reverse Transcription kit (ThermoFisher, UK) following manufacturer’s protocol. cDNA samples (2 μl) were combined with primers and PowerUp SYBR Green Mastermix (ThermoFisher, UK) following manufacturer’s instructions. RT-qPCR reactions were run in duplicate on a BioRad CFX thermal cycler (BioRad, UK) according to the following protocol: 50 ^o^C for 2 minutes, 95 ^o^C for 2 minutes and 40 cycles of 95 ^o^C 15 seconds and 60 ^o^C for 1 minute. Melt curve analysis was included to ensure amplification of a single PCR product and non-reverse transcribed controls were used to confirm no contamination with viral or genomic DNA. Data was analysed using the Livak method (2^-ΔΔCt^) using *β-actin* as a housekeeping gene and normalising to the relevant controls for each experiment [64]. High throughput dynamic array RT-qPCR was also carried out using a Biomark HD (Fluidigm, UK) in the 96.96 format. In this case data was analysed using the Livak method (2^-ΔΔCt^) using *Gapdh* as a housekeeping gene and normalising to the Ad-CMV-Null treated cells. Primer pairs used are provided in **S1 Table**.

### Immunocytochemistry

Cells grown on poly-l-lysine/laminin coated 8 well MilliCell EZ slides were fixed in ice cold 4% PFA (Sigma-Aldrich, UK) for 15 minutes, permeabilized with PBS containing 0.3% Triton-X (Sigma-Aldrich, UK) for 10 minutes and blocked in PBS containing 0.1% Tween20 (PBST) with 10% normal goat serum (NGS) (ThermoFisher, UK) and 0.3M glycine (Sigma-Aldrich, UK) for 1 hour. Cells were then incubated with primary antibodies in PBST containing 10% NGS overnight at 4 ^o^C in a humidified chamber. The following day, cells were washed in PBST and incubated in secondary antibody diluted in PBST containing 10% NGS for 1 hour at room temperature. Cells were washed with 3 times with PBST, twice with PBS and mounted with ProLong Gold mounting reagent with DAPI (ThermoFisher, UK) which was allowed to cure for 24 hours prior to imaging. Fluorescent slides were imaged on a Zeiss AXIO Observser.A1 using a 10x or 20x objective. Higher magnification images were acquired using an Olympus IX83 inverted microscope using the 60x objective with a Z optical spacing of 0.2 μm. Where appropriate, brightness/contrast for individual fluorescence channels was adjusted equally between experimental samples and controls. Images were analysed and quantified using ImageJ (NIH, USA). The antibodies used in these investigations are provided in **S2 Table**.

### Statistical analysis

A minimum of 3 biological replicates was included for each experiment unless otherwise stated in the figure legend. Statistical analysis was conducted using mean values from each biological replicate and did not recognise technical replicates (fields of view/duplicate PCR reactions) as individual n-numbers. For RT-qPCR data statistical analysis was conducted on untransformed ΔCt values. Statistical analysis was carried out using GraphPad Prism 8 to perform unpaired t-test’s for comparisons between 2 groups with correction for multiple comparisons where appropriate, or ANOVA followed by Tukey’s post-hoc analysis for comparison between 3 or more groups. A probability (P) of < 0.05 was regarded as statistically significant and P values and n numbers are specified in the figure legends. All data are presented as mean ± S.D.

## Supporting information

S1 Fig Immunofluorescence of starting cardiomyocyte population. (**A**) Representative phase contrast and immunofluorescence images of cardiomyocytes on day 0 of transduction (Scale bar = 200 μm). (**B**) Quantification of cardiomyocytes (cTnT+/VIM-) and non-myocytes (VIM+/cTnT-) as a percentage of total population (n = 8 fields). (**C**) Co-expression of NKX2-5 exclusively in cTnT positive cells (Scale bar = 200 μm). (B) Data presented as mean percentage ± S.D.
(DOCX)

S2 Fig Cardiac gene expression in control vector treated cardiomyocytes. (**A**) Gene expression of *Myh6* and *Myh7* in cardiomyocytes treated with Ad-CMV-Null (n = 3). Data are presented as mean ± S.D. one-way ANOVA with Tukey’s post hoc analysis. No statistically significant differences were observed.
(DOCX)

S3 Fig SOX2 expression level correlated with cardiomyocyte phenotype. Immunostaining of SOX2 and cTnT in Ad-CMV-MKOS transduced cardiomyocytes (3 days post transduction). SOX2 high was defined as cells with a nuclear SOX2 fluorescence signal greater than the median fluorescence intensity of the SOX2 positive population and SOX2 low defined as signals below this median. Representative image from n = 4 replicates, 4–6 fields per replicate. (Scale bar = 100 μm).
(DOCX)

S4 Fig Change of cTnT expression in labelled mouse cardiomyocytes. (**A**) Immunofluorescence of αMHC-Cre-tdTomato cardiomyocytes 3 days post transduction with either Ad-CMV-Null or Ad-CMV-MKOS (scale bars = 50 μm). Representative image from n = 2 replicates, 4–6 fields per replicate. (**B**) Quantification of the percentage of transduced cardiomyocytes (SOX2+tdTomato+ cells) with absent cTnT expression (n = 2 replicates, 4–6 fields per replicate).
(DOCX)

S5 Fig Immunofluorescence of Ki67 in OSKM transduced mouse cardiomyocytes. (**A**) Ki67 expression in αMHC-Cre-tdTomato cardiomyocytes 3 days post transduction (Scale bar = 100 μm). (**B**) Quantification of Ki67+ nuclei and tdTomato+ Ki67+ cells (n = 2 replicates/4 fields per replicate). (**c**) Expression of Ki67 in cTnT- tdTomato+ cells 3 days post transduction (Scale bar = 50 μm). Data are presented as mean ± S.D. (**B**) Unpaired t-tests, no statistically significant differences identified.
(DOCX)

S6 Fig Immunofluorescence of ECad and NKX2-5 in cardiomyocytes treated with control vector. Representative image from n = 2 replicates, 4 fields per replicate (Scale bars = 100 μm).
(DOCX)

S7 Fig Co-expression of ECad and tdTomato in labelled mouse cardiomyocytes. (**A**) Presence of ECad+ tdTomato+ cells in αMHC-Cre-tdTomato cardiomyocytes 5 days post transduction (Scale bar = 100 μm). (**B**) High magnification of ECad+ cells with orthogonal view to confirm co-localisation with tdTomato (Scale bar = 50 μm).
(DOCX)

S8 Fig Morphology of cardiomyocytes in ESC media. (**A**) Phase contrast microscopy of cardiomyocytes days 3–15 post transduction with Ad-CMV-MKOS in the presence of ESC media (Scale bar = 200 μm). (**B**) Lack of ESC-like colonies in cardiomyocytes transduced with Ad-CMV-MKOS day 20 post transduction (Scale bar = 400 μm). Representative images from n = 3 repeats/4 fields per repeat.
(DOCX)

S1 Table Primer pairs utilised in RT-qPCR investigations. (DOCX)

S2 Table Antibodies utilised in immunocytochemistry investigations. (DOCX)

S1 Video Ad-CMV-Null treated cardiomyocytes day 3 post transduction. (MP4)

S2 Video Ad-CMV-MKOS treated cardiomyocytes day 3 post transduction. (MP4)

S3 Video Ad-CMV-Null treated cardiomyocytes day 10 post transduction. (MP4)

S4 Video Ad-CMV-MKOS treated cardiomyocytes day 10 post transduction. (MP4)

S5 Video Ad-CMV-Null treated cardiomyocytes day 20 post transduction. (MP4)

S6 Video Ad-CMV-MKOS treated cardiomyocytes day 20 post transduction. (MP4)
