# Supplementary material for: Transient reprogramming of postnatal cardiomyocytes to a dedifferentiated state
Source: PLoS One. 2021 May 5;16(5):e0251054. doi: 10.1371/journal.pone.0251054 (PMC8099115; doi:10.1371/journal.pone.0251054)
Supplement: S1 Fig — (A) Representative phase contrast and immunofluorescence images of cardiomyocytes on day 0 of transduction (Scale bar = 200 μm). (B) Quantification of cardiomyocytes (cTnT+/VIM-) and non-myocytes (VIM+/cTnT-) as a percentage of total population (n = 8 fields). (C) Co-expression of NKX2-5 exclusively in cTnT positive cells (Scale bar = 200 μm). (B) Data presented as mean percentage ± S.D. (DOCX) [file pone.0251054.s001.docx]

**
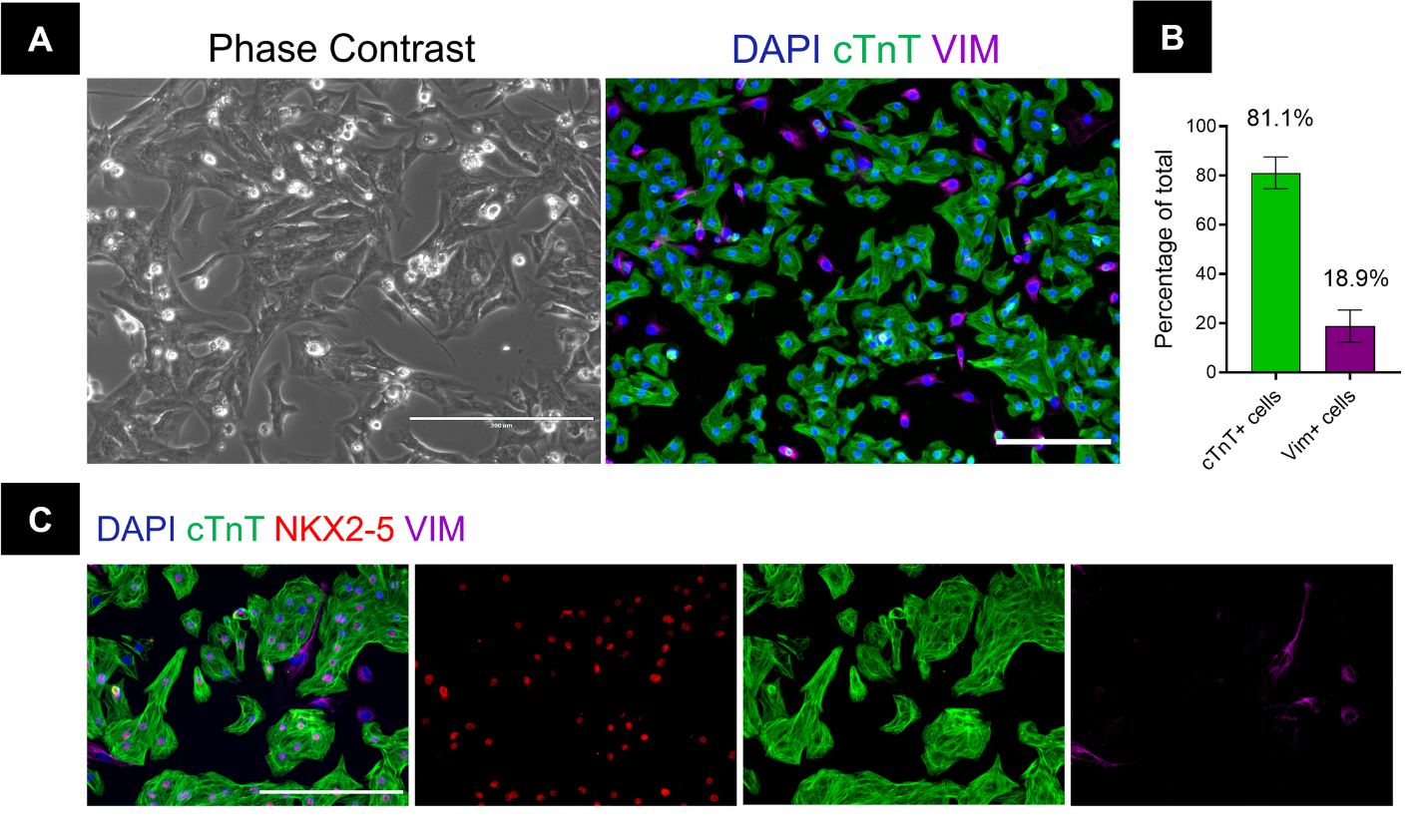
**

**S1 Fig: Immunofluorescence of starting cardiomyocyte population.** (**A**) Representative phase contrast and immunofluorescence images of cardiomyocytes on day 0 of transduction (Scale bar = 200 µm). (**B**) Quantification of cardiomyocytes (cTnT+/VIM-) and non-myocytes (VIM+/cTnT-) as a percentage of total population (n=8 fields). (**C**) Co-expression of NKX2-5 exclusively in cTnT positive cells (Scale bar = 200 µm).

(B) Data presented as mean percentage ± S.D.
