# Supplementary material for: Transient reprogramming of postnatal cardiomyocytes to a dedifferentiated state
Source: PLoS One. 2021 May 5;16(5):e0251054. doi: 10.1371/journal.pone.0251054 (PMC8099115; doi:10.1371/journal.pone.0251054)
Supplement: S2 Fig — (A) Gene expression of Myh6 and Myh7 in cardiomyocytes treated with Ad-CMV-Null (n = 3). Data are presented as mean ± S.D. one-way ANOVA with Tukey’s post hoc analysis. No statistically significant differences were observed. (DOCX) [file pone.0251054.s002.docx]

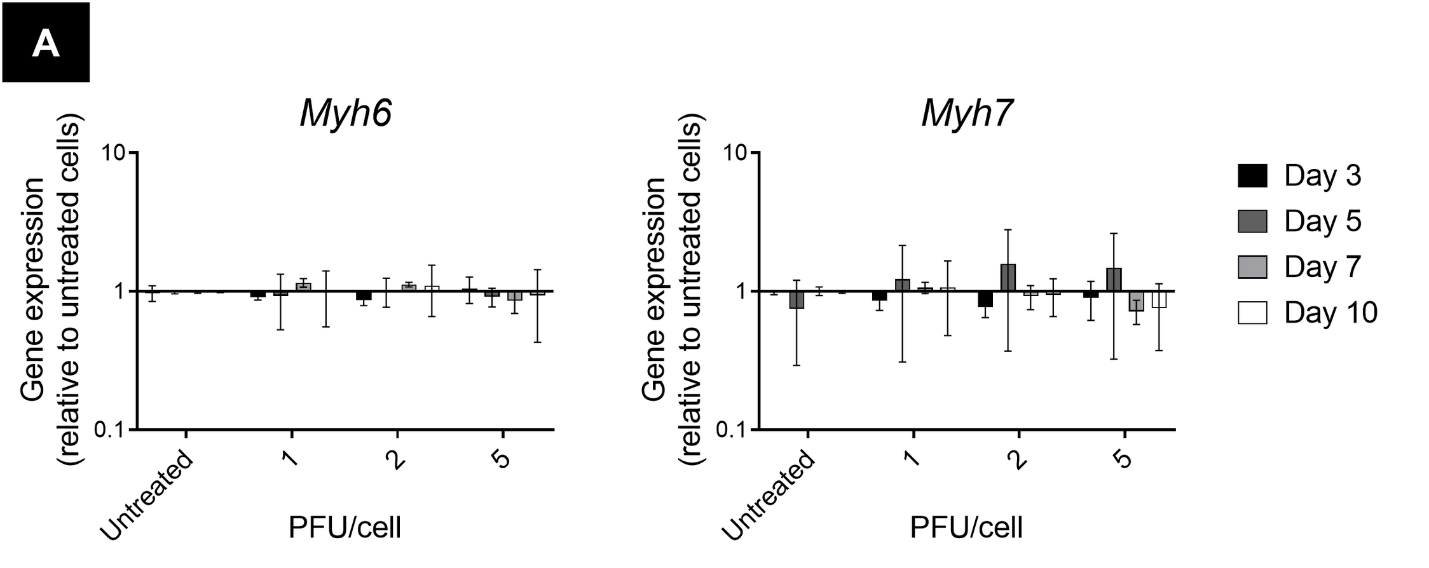


**S2 Fig**: **Cardiac gene expression in control vector treated cardiomyocytes.** (**A**) Gene expression of *Myh6* and *Myh7* in cardiomyocytes treated with Ad-CMV-Null (n=3).

Data are presented as mean ± S.D. one-way ANOVA with Tukey’s post hoc analysis. No statistically significant differences were observed.
