# Supplementary material for: Transient reprogramming of postnatal cardiomyocytes to a dedifferentiated state
Source: PLoS One. 2021 May 5;16(5):e0251054. doi: 10.1371/journal.pone.0251054 (PMC8099115; doi:10.1371/journal.pone.0251054)
Supplement: S3 Fig — Immunostaining of SOX2 and cTnT in Ad-CMV-MKOS transduced cardiomyocytes (3 days post transduction). SOX2 high was defined as cells with a nuclear SOX2 fluorescence signal greater than the median fluorescence intensity of the SOX2 positive population and SOX2 low defined as signals below this median. Representative image from n = 4 replicates, 4–6 fields per replicate. (Scale bar = 100 μm). (DOCX) [file pone.0251054.s003.docx]

**
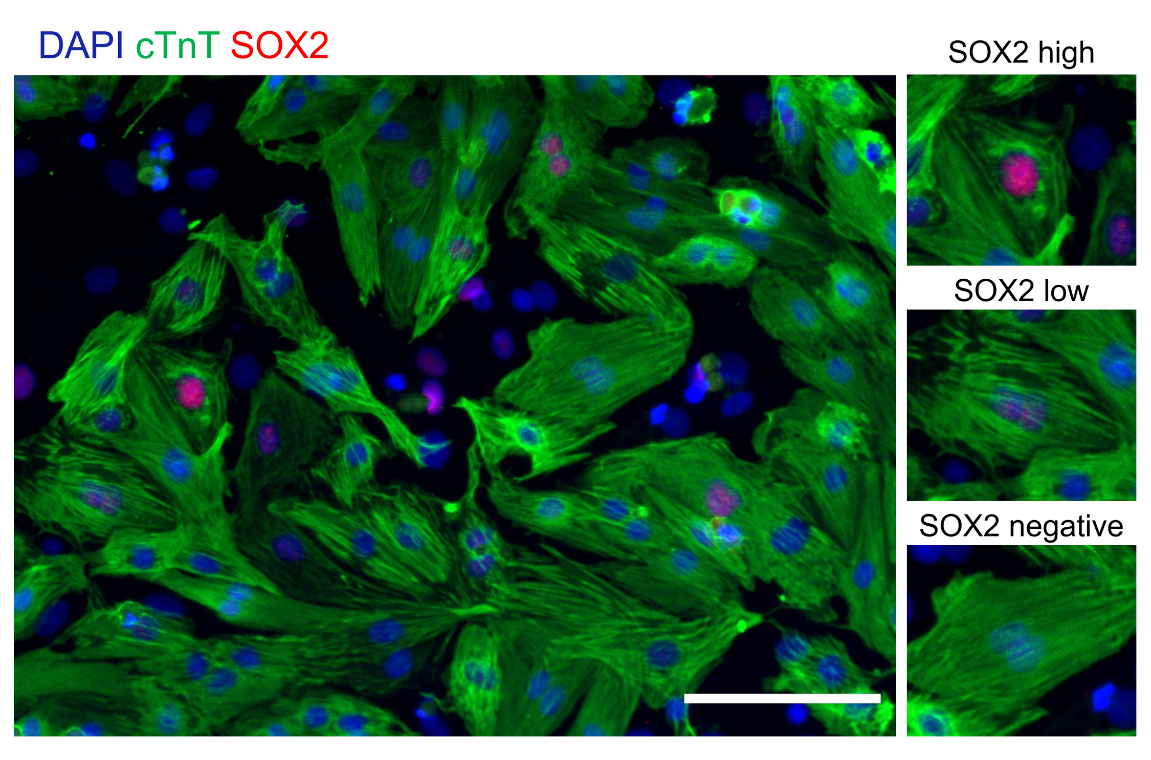
**

**S3 Fig: SOX2 expression level correlated with cardiomyocyte phenotype**. Immunostaining of SOX2 and cTnT in Ad-CMV-MKOS transduced cardiomyocytes (3 days post transduction). SOX2 high was defined as cells with a nuclear SOX2 fluorescence signal greater than the median fluorescence intensity of the SOX2 positive population and SOX2 low defined as signals below this median. Representative image from n=4 replicates, 4-6 fields per replicate. (Scale bar = 100 µm).
