# Supplementary material for: Transient reprogramming of postnatal cardiomyocytes to a dedifferentiated state
Source: PLoS One. 2021 May 5;16(5):e0251054. doi: 10.1371/journal.pone.0251054 (PMC8099115; doi:10.1371/journal.pone.0251054)
Supplement: S4 Fig — (A) Immunofluorescence of αMHC-Cre-tdTomato cardiomyocytes 3 days post transduction with either Ad-CMV-Null or Ad-CMV-MKOS (scale bars = 50 μm). Representative image from n = 2 replicates, 4–6 fields per replicate. (B) Quantification of the percentage of transduced cardiomyocytes (SOX2+tdTomato+ cells) with absent cTnT expression (n = 2 replicates, 4–6 fields per replicate). (DOCX) [file pone.0251054.s004.docx]

**
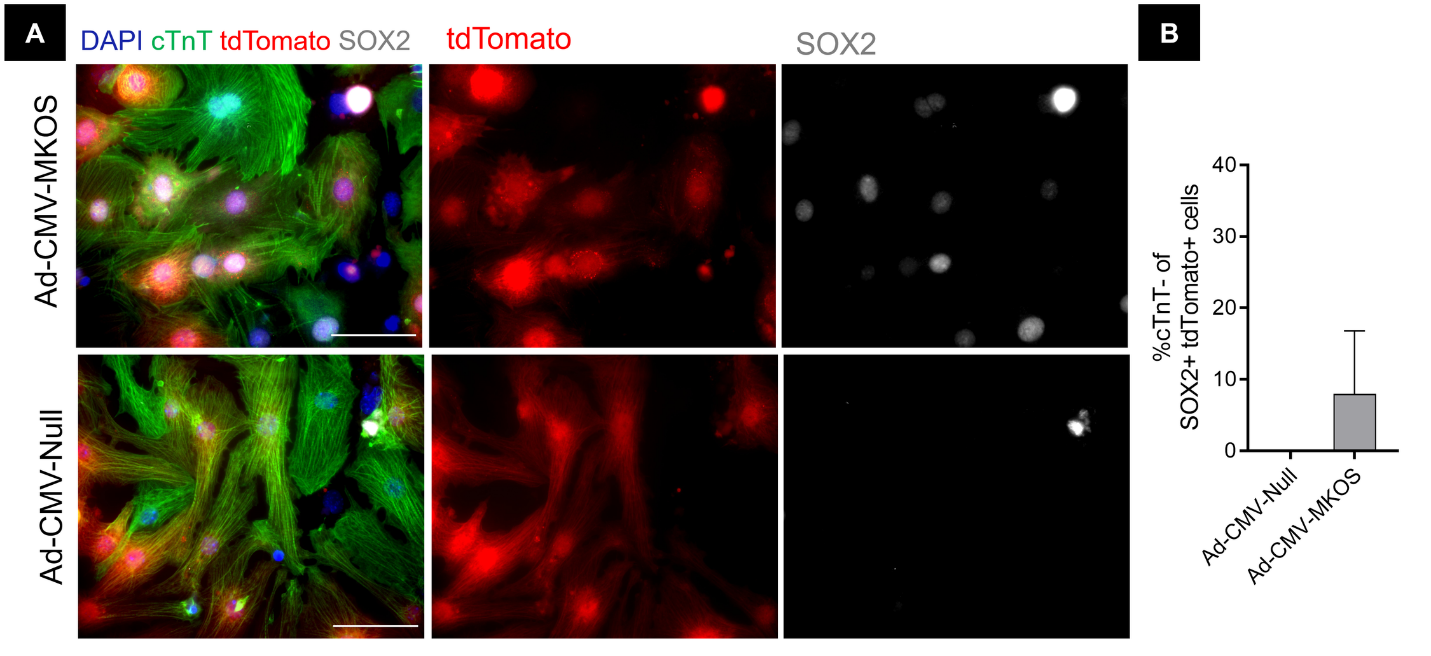
**

**S4 Fig**: **Change of cTnT expression in labelled mouse cardiomyocytes** (**A**) Immunofluorescence of αMHC-Cre-tdTomato cardiomyocytes 3 days post transduction with either Ad-CMV-Null or Ad-CMV-MKOS (scale bars = 50 µm). Representative image from n=2 replicates, 4-6 fields per replicate. (**B**) Quantification of the percentage of transduced cardiomyocytes (SOX2+tdTomato+ cells) with absent cTnT expression (n=2 replicates, 4-6 fields per replicate).
