# Supplementary material for: Transient reprogramming of postnatal cardiomyocytes to a dedifferentiated state
Source: PLoS One. 2021 May 5;16(5):e0251054. doi: 10.1371/journal.pone.0251054 (PMC8099115; doi:10.1371/journal.pone.0251054)
Supplement: S5 Fig — (A) Ki67 expression in αMHC-Cre-tdTomato cardiomyocytes 3 days post transduction (Scale bar = 100 μm). (B) Quantification of Ki67+ nuclei and tdTomato+ Ki67+ cells (n = 2 replicates/4 fields per replicate). (c) Expression of Ki67 in cTnT- tdTomato+ cells 3 days post transduction (Scale bar = 50 μm). Data are presented as mean ± S.D. (B) Unpaired t-tests, no statistically significant differences identified. (DOCX) [file pone.0251054.s005.docx]

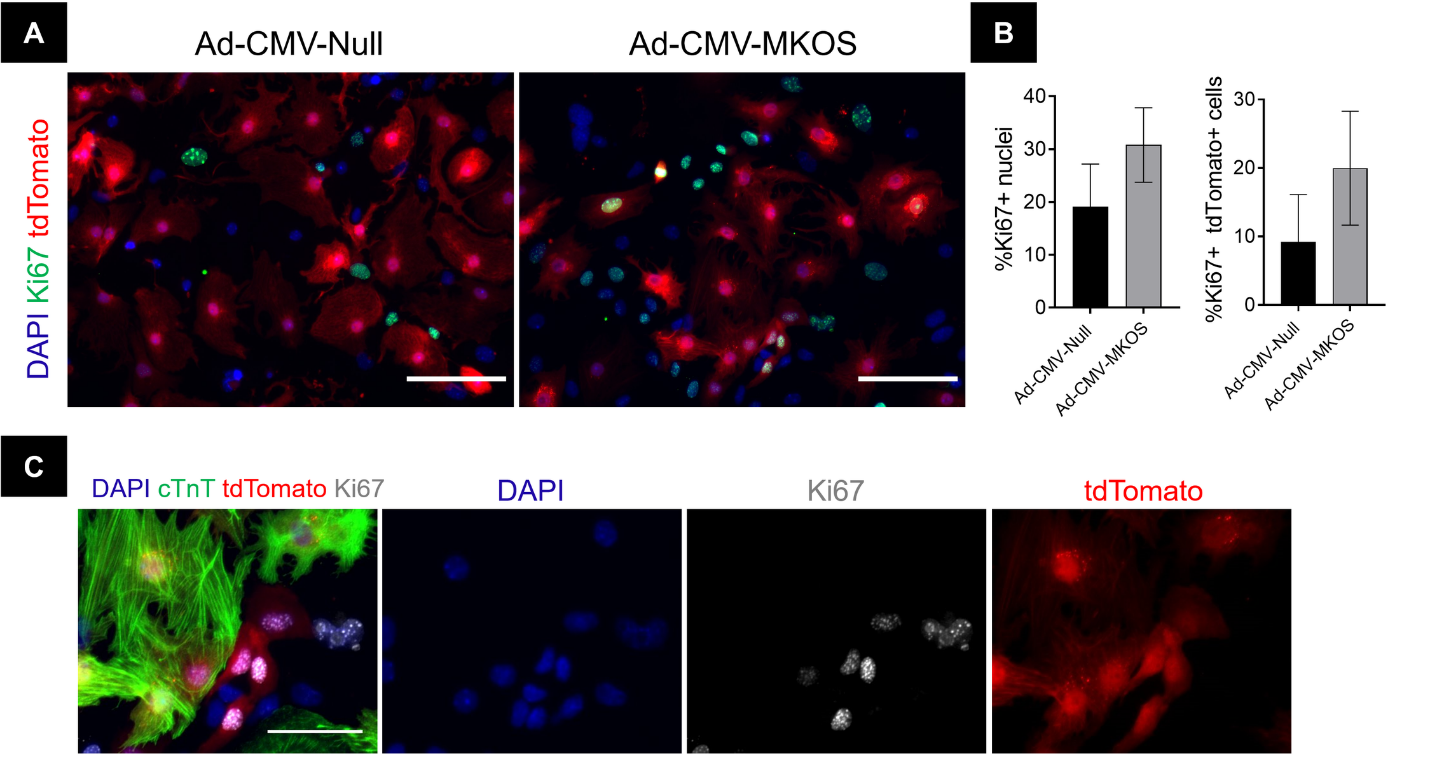


**S5 Fig: Immunofluorescence of Ki67 in OSKM transduced mouse cardiomyocytes** (**A**) Ki67 expression in αMHC-Cre-tdTomato cardiomyocytes 3 days post transduction (Scale bar = 100 µm). (**B**) Quantification of Ki67+ nuclei and tdTomato+ Ki67+ cells (n=2 replicates/4 fields per replicate). (**c**) Expression of Ki67 in cTnT- tdTomato+ cells 3 days post transduction (Scale bar = 50 µm).

Data are presented as mean ± S.D. (**B**) Unpaired t-tests, no statistically significant differences identified.
