# Supplementary material for: Transient reprogramming of postnatal cardiomyocytes to a dedifferentiated state
Source: PLoS One. 2021 May 5;16(5):e0251054. doi: 10.1371/journal.pone.0251054 (PMC8099115; doi:10.1371/journal.pone.0251054)
Supplement: S6 Fig — Representative image from n = 2 replicates, 4 fields per replicate (Scale bars = 100 μm). (DOCX) [file pone.0251054.s006.docx]

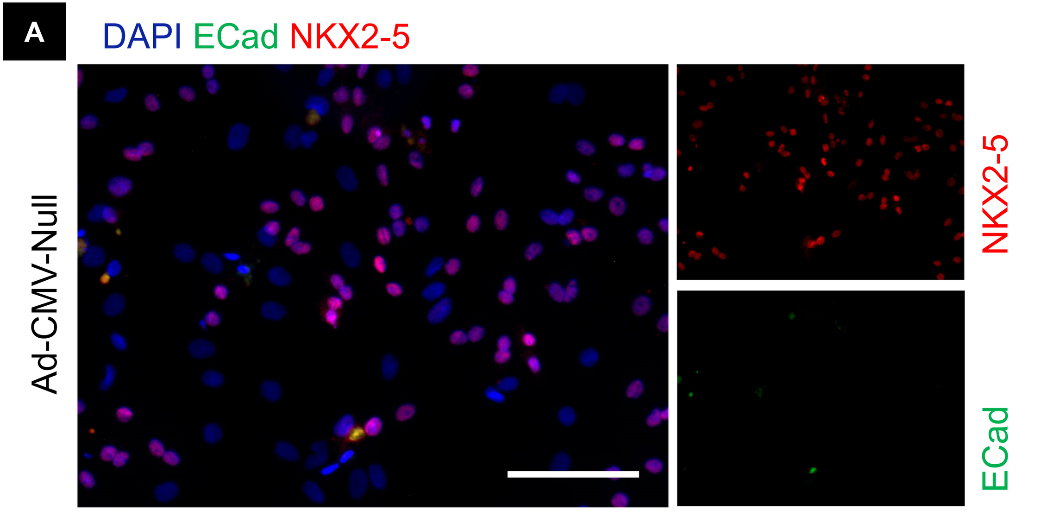


**S6 Fig: Immunofluorescence of ECad and NKX2-5 in cardiomyocytes treated with control vector.** Representative image from n=2 replicates, 4 fields per replicate (Scale bars = 100 µm).
