# Supplementary material for: Transient reprogramming of postnatal cardiomyocytes to a dedifferentiated state
Source: PLoS One. 2021 May 5;16(5):e0251054. doi: 10.1371/journal.pone.0251054 (PMC8099115; doi:10.1371/journal.pone.0251054)
Supplement: S7 Fig — (A) Presence of ECad+ tdTomato+ cells in αMHC-Cre-tdTomato cardiomyocytes 5 days post transduction (Scale bar = 100 μm). (B) High magnification of ECad+ cells with orthogonal view to confirm co-localisation with tdTomato (Scale bar = 50 μm). (DOCX) [file pone.0251054.s007.docx]

**
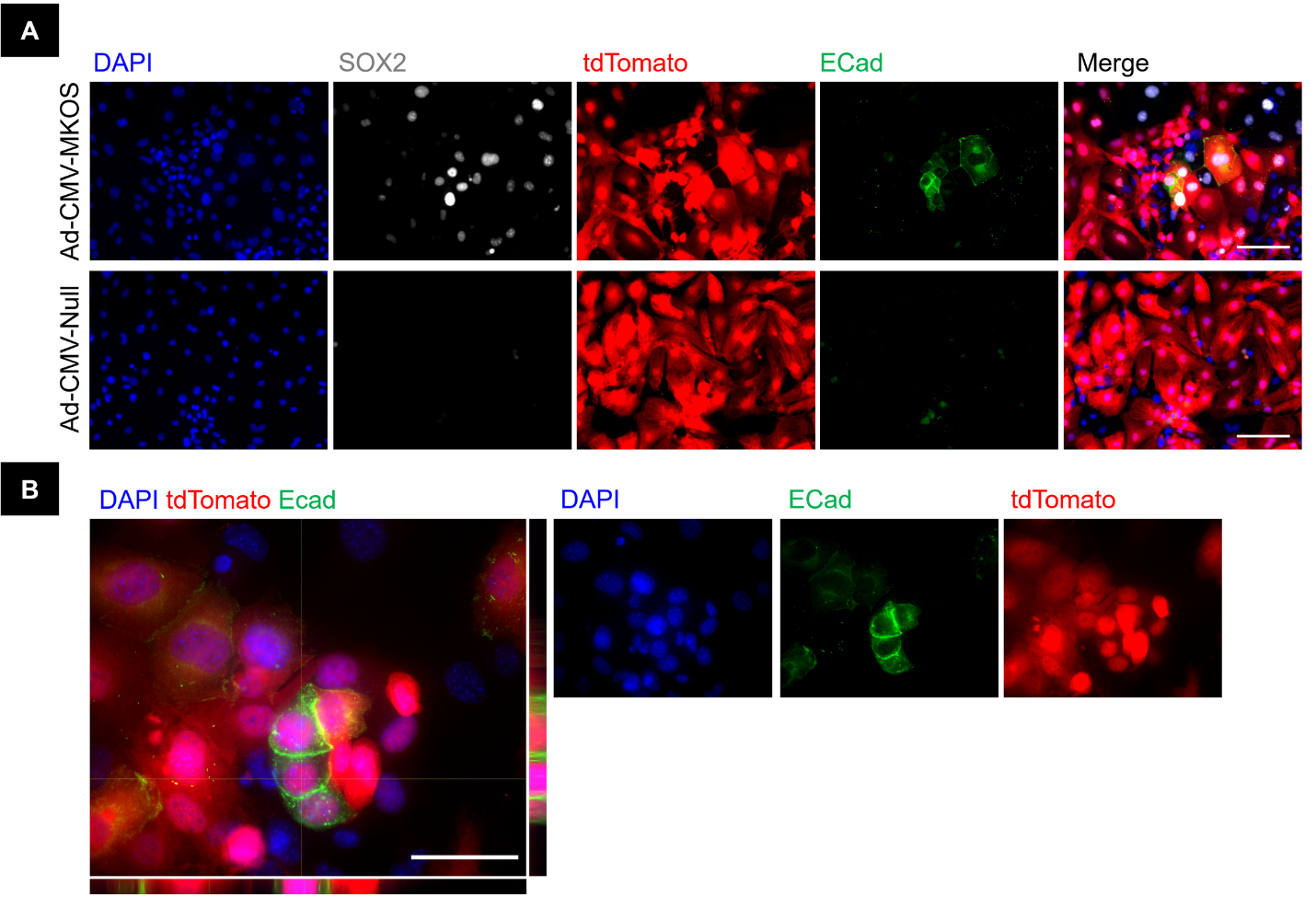
**

**S7 Fig: Co-expression of ECad and tdTomato in labelled mouse cardiomyocytes** (**A**) Presence of ECad+ tdTomato+ cells in αMHC-Cre-tdTomato cardiomyocytes 5 days post transduction (Scale bar = 100 µm). (**B**) High magnification of ECad+ cells with orthogonal view to confirm co-localisation with tdTomato (Scale bar = 50 µm).
