# Supplementary material for: Transient reprogramming of postnatal cardiomyocytes to a dedifferentiated state
Source: PLoS One. 2021 May 5;16(5):e0251054. doi: 10.1371/journal.pone.0251054 (PMC8099115; doi:10.1371/journal.pone.0251054)
Supplement: S8 Fig — (A) Phase contrast microscopy of cardiomyocytes days 3–15 post transduction with Ad-CMV-MKOS in the presence of ESC media (Scale bar = 200 μm). (B) Lack of ESC-like colonies in cardiomyocytes transduced with Ad-CMV-MKOS day 20 post transduction (Scale bar = 400 μm). Representative images from n = 3 repeats/4 fields per repeat. (DOCX) [file pone.0251054.s008.docx]

**
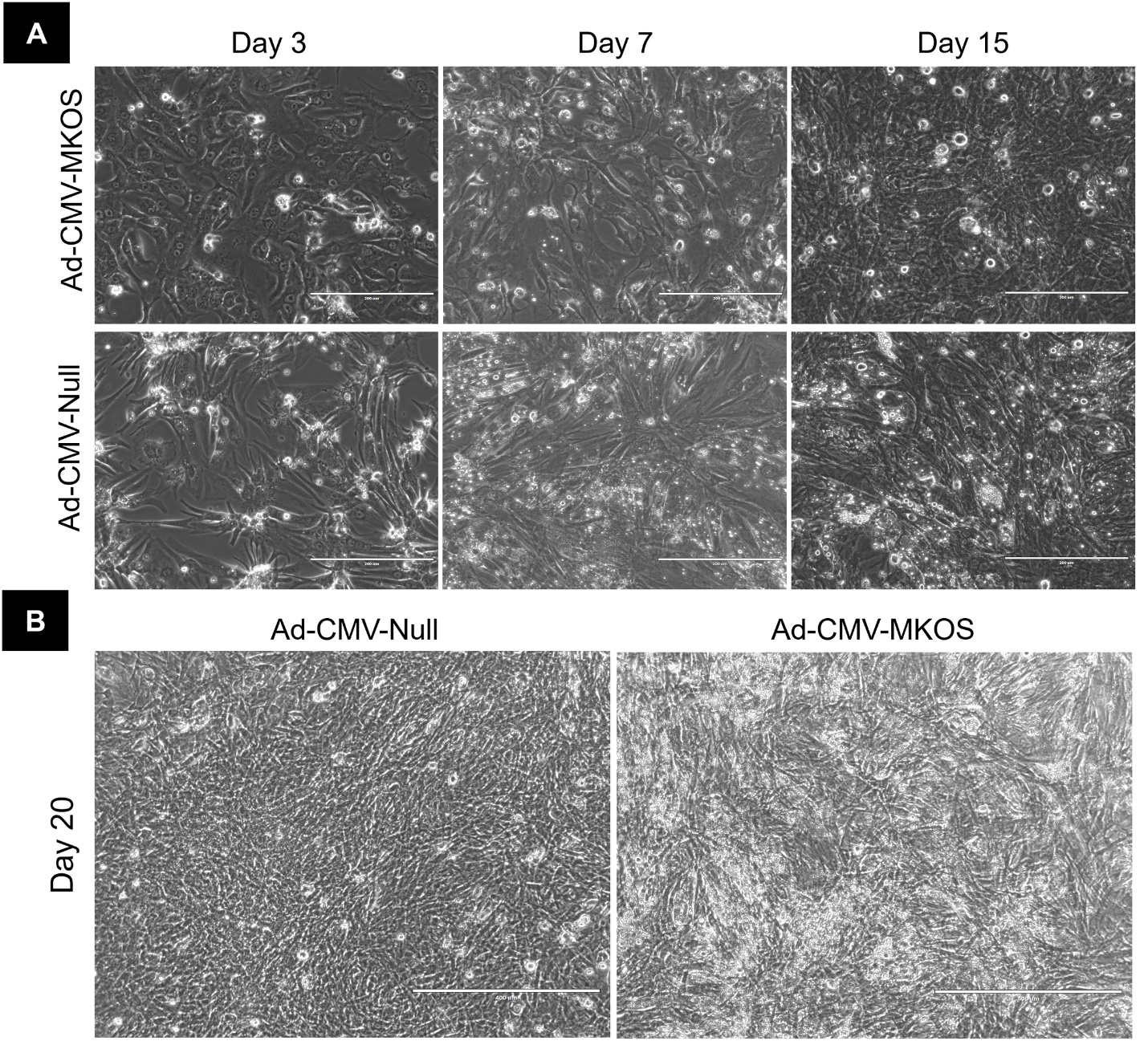
**

**S8 Fig: Morphology of cardiomyocytes in ESC media** (**A**) Phase contrast microscopy of cardiomyocytes days 3-15 post transduction with Ad-CMV-MKOS in the presence of ESC media (Scale bar = 200 µm). (**B**) Lack of ESC-like colonies in cardiomyocytes transduced with Ad-CMV-MKOS day 20 post transduction (Scale bar = 400 µm). Representative images from n=3 repeats/4 fields per repeat.
