# Supplementary material for: Transient reprogramming of postnatal cardiomyocytes to a dedifferentiated state
Source: PLoS One. 2021 May 5;16(5):e0251054. doi: 10.1371/journal.pone.0251054 (PMC8099115; doi:10.1371/journal.pone.0251054)
Supplement: S1 Table — (DOCX) [file pone.0251054.s009.docx]

**S1 Table:** Primer pairs utilised in RT-qPCR investigations.

| **Gene symbol** | **Forward Primer (5'->3')** | **Reverse Primer (5'->3')** |
| --- | --- | --- |
| Oct3/4 (total) | TGAGAACCTTCAGGAGATATGCAA | CTCAATGCTAGTTCGCTTTCTCTTC |
| Oct3/4 (endogenous) | TCTTTCCACCAGGCCCCCGGCTC | TGCGGGCGGACATGGGGAGATCC |
| Sox2 (total) | GGTTACCTCTTCCTCCCACTCCAG | TCACATGTGCGACAGGGGCAG |
| Klf4 (total) | CAGTGGTAAGGTTTCTCGCC | GCCACCCACACTTGTGACTA |
| cMyc (total) | CAGAGGAGGAACGAGCTGAAGCGC | TTATGCACCAGAGTTTCGAAGCTGTTCG |
| Actb | GACCTCTATGCCAACACAGT | AGTACTTGCGCTCAGGAGGA |
| Alpl | ATCGGACCCTGCCTTACCAA | GGAGACGCCCATACCATCTC |
| Bmp4 | TGGACACCTCATCACACGACTA | GCGACGGCAGTTCTTATTCTTC |
| Ccna2 | AGTGCCGCTGTCTCTTTACC | GGGGTGATTCAAAACTACCATCC |
| Ccnd1 | TCAAGTGTGACCCGGACTG | GACCAGCTTCTTCCTCCACTT |
| Cdh1 | TTCAACCCAAGCACGTACCA | CAGAATGCCCTCGTTGGTCT |
| Cdkn1a | CACGGCTCAGTGGACCAGAA | ACTGGAGCTGCCTGAGGTAGGA |
| Cdkn2a | CGTACCCCGATACAGGTGATG | ACCAGAGTGTCTAGGAAGCCC |
| Dppa4 | CTTTCCCAGATCAAATGCCCG | TCTTCCCCAGGTTCCGTCTT |
| Epcam | CGGGGATTGTTGTCCTGGTT | CCCATCTCCTTTATCTCAGCCTTC |
| Esrrb | GGGATGCTGAAGGAAGGCGT | TTAGCAGGCGGGGAAATCTG |
| Fut4 | GCTGTCTATCGCCGCTACTT | TGCCAAGTTGTGGATGCTCT |
| Gapdh | ACCACAGTCCATGCCATCAC | TCCACCACCCTGTTGCTGTA |
| Gata4 | CTCTATCACAAGATGAACGGCATCAA | TCTGGCAGTTGGCACAGGAGAG |
| Isl1 | CTGCAAATGGCAGCCGAGC | GGTCTTCTCGGGCTGTTTGT |
| Kit | CTCCAACGATGTGGGCAAGA | GGGCCTGGATTTGCTCTTTG |
| Mef2c | AGGCACCAGCGCAGGGAATG | CCACCGGGGTAGCCAATGACT |
| Mesp1 | CATTTAAGCCCGGTTGCCTG | TGCTGAAGAGCGGAGACGAG |
| Mesp2 | AACAAGACTGGGCACTGGAC | CTGGAGACACAGAAAGACTCTGG |
| Myh6 | TAACCGGAGTTTAAGAGTGACAGG | TAGGCGCTCCTTCTCTGACT |
| Myh7 | CTGGCACCGTGGACTACAAT | GCCCTTGTCTACAGGTGCAT |
| Nkx2-5 | ACCGCCCCTACATTTTATCCG | CACAGCTCTTTCTTATCCGCCC |
| Pecam1 | CTGCCAGTCAGTAAATGGGAC | CTTCATCCACCGGGGCTATT |
| Sox17 | GGCACGGAACCCAACCAGC | CAGTCGTGTCCCTGGTAGGGAAGAC |
| Tbx5 | GGTCCGTAACTGGTAAAG | ATTTTCGTCTGCTTTCAC |
| Tbxt (T) | AGAATGAGGAGATTACGGCCC | ATTGGGAATATCCCGGCTGC |
| Vim | GCGAGAGAAATTGCAGGAGGA | CGTTCAAGGTCAAGACGTGC |
