# Supplementary material for: Transient reprogramming of postnatal cardiomyocytes to a dedifferentiated state
Source: PLoS One. 2021 May 5;16(5):e0251054. doi: 10.1371/journal.pone.0251054 (PMC8099115; doi:10.1371/journal.pone.0251054)
Supplement: S2 Table — (DOCX) [file pone.0251054.s010.docx]

**S2 Table:** Antibodies utilised in immunocytochemistry investigations.

| **Primary antibodies** | | | |
| --- | --- | --- | --- |
| **Antibody** | **Species** | **Supplier** (Catalogue number) | **Dilution used** |
| Anti-Sox2 | Rabbit | Abcam (ab97959) | 1:500 |
| Anti-Oct3/4 | Rabbit | Abcam (ab19857) | 1:250 |
| Anti-Oct3/4 | Rat | ThermoFisher (14-5841-82) | 1:250 |
| Anti-Nanog | Rabbit | Abcam (ab80892) | 1:200 |
| Anti-Nanog | Rabbit | Abcam (ab106465) | 1:200 |
| Anti-Cardiac TroponinT | Mouse | Abcam (ab8295) | 1:500 |
| Anti-Nkx2-5 | Rabbit | ProteinTech (13921-1-AP) | 1:300 |
| Anti-ECadherin | Mouse | Abcam (ab76055) | 1:200 |
| Anti-Ki67 | Rabbit | Abcam (ab15580) | 1:800 |
| Anti-Vimentin | Chicken | Abcam (ab24525) | 1:1000 |
| **Secondary antibodies** | | | |
| **Antibody** | **Species** | **Supplier (Catalogue number)** | **Dilution used** |
| Anti-Mouse Alexa Fluor 488 | Goat | ThermoFisher (A11001) | 1:500 |
| Anti-Rabbit Alexa Fluor 594 | Goat | ThermoFisher (A11012) | 1:500 |
| Anti-Rat Alexa Fluor 647 | Goat | ThermoFisher (A21247) | 1:300 |
| Anti-Chicken Alexa Fluor 647 | Goat | ThermoFisher (A21449) | 1:1000 |
